# Three New Benzophenone Derivatives from *Selaginella tamariscina*

**DOI:** 10.3390/molecules28124582

**Published:** 2023-06-06

**Authors:** Jiayin Long, Qingqing Mao, Yujie Peng, Lei Liu, Yin Hong, Honglin Xiang, Ming Ma, Hui Zou, Junwei Kuang

**Affiliations:** 1 Key Laboratory of Study and Discovery of Small Targeted Molecules of Hunan Province, School of Medicine, Hunan Normal University, Changsha 410013, China; 202020191549@hunnu.edu.cn (J.L.); 202020191550@hunnu.edu.cn (Q.M.); 202130192065@hunnu.edu.cn (Y.P.); 201930192015@smail.hunnu.edu.cn (L.L.); 01930192016@hunnu.edu.cn (Y.H.); 13828@hunnu.edu.cn (H.X.); 2 Key Laboratory of Phytochemical R&D of Hunan Province, Key Laboratory of Chemical Biology & Traditional Chinese Medicine Research of Ministry of Education, College of Chemistry and Chemical Engineering, Hunan Normal University, Changsha 410081, China; mingma@hunnu.edu.cn

**Keywords:** *Selaginella*, *Selaginella tamariscina*, benzophenone, selagibenzophenones D-F, cytotoxicity, NO inhibitory effects

## Abstract

Six compounds including three new benzophenones, selagibenzophenones D-F (**1**–**3**), two known selaginellins (**4**–**5**) and one known flavonoid (**6**), were isolated from *Selaginella tamariscina*. The structures of new compounds were established by 1D-, 2D-NMR and HR-ESI-MS spectral analyses. Compound **1** represents the second example of diarylbenzophenone from natural sources. Compound **2** possesses an unusual biphenyl-bisbenzophenone structure. Their cytotoxicity against human hepatocellular carcinoma HepG2 and SMCC-7721 cells and inhibitory activities on lipopolysaccharide-induced nitric oxide (NO) production in RAW264.7 cells were evaluated. Compound **2** showed moderate inhibitory activity against HepG2 and SMCC-7721 cells, and compounds **4** and **5** showed moderate inhibitory activity to HepG2 cells. Compounds **2** and **5** also exhibited inhibitory activities on lipopolysaccharide-induced nitric oxide (NO) production.

## 1. Introduction

The genus *Selaginella* (Selaginellaceae) comprises about 700 species and about 70 species were widely dispersed across the area south of the Yangtze River [1]. In Hunan province, more than ten species, such as *Selaginella tamariscina, Selaginella Pulvinata* and *Selaginella uncinata* were found distributed in various regions. It was estimated that the genus Selaginella has existed on earth for over 400 million years. Selaginella species not only plays an important role in evolutionary history as the living fossils of vascular plants, but also occupied a pivotal position in the history of medicine. The research history of chemical constituents of *Sleaginella* can be traced back to 1971. Okigawa et al. reported three flavonoids from *S. tamariscina* in 1971 [2]. Afterwards, a large number of flavonoids and phenylpropanoids were isolated from this genus. Meanwhile, the good pharmacological effects of *Selaginella* species also attracted considerable attention. The extract and isolates from *Selaginella* showed extensive pharmacological effects including anticancer [3], anti-inflammatory [4], antivirus [5], cardiovascular protection [6], anti-hyperglycemic [7].

*S. tamariscina* was included in the 2020 edition of the Chinese pharmacopoeia [8], which was used as a Traditional Chinese Medicine (TCM) to treat chronic hepatitis, inflammation, and cancer. A number of studies demonstrated that phytochemicals from *S. tamariscina* exhibited anticancer [9,10,11,12], anti-inflammatory [13,14], antibacterial [15], and PDE4 inhibition effects [16]. Phytochemical studies have shown that the main chemical constituents of *S. tamariscina* were flavonoids [17,18,19], selaginellins [20,21], and lignans [22]. Benzophenone derivatives were important bioactive natural products and showed anticancer and anti-inflammatory effects [23,24]. Liu et al. [25] reported the first natural triarylbenzophenone from *S. pulvinata* and we reported the second natural triarylbenzophenone derivative and the first natural biarylbenzophenone derivative from *S. tamariscina* in our previous research [26,27]. Selaginellins represent a type of characteristic constituent of *Selaginella*. So far, over one hundred selaginellins with different polyphenolic skeletons have been reported from this genus [1]. A number of these selaginellins showed good anticancer activity [28] and PDE4 inhibitory activity [29]. Therefore, it is important to explore the novel natural products from *S. tamariscina* and their potential pharmacological effects. In the present study, we report the isolation and structural elucidation of three new benzophenone derivatives and three known compounds including two selaginellins and one flavonoid. In addition, compounds **1**–**6** were evaluated for their cytotoxicity against human hepatocellular carcinoma HepG2 and SMCC-7721 cells and for their inhibitory activities on lipopolysaccharide-induced nitric oxide (NO) production in RAW264.7 cells.

## 2. Results and Discussion

### 2.1. Structure Determination

**Figure 1:**
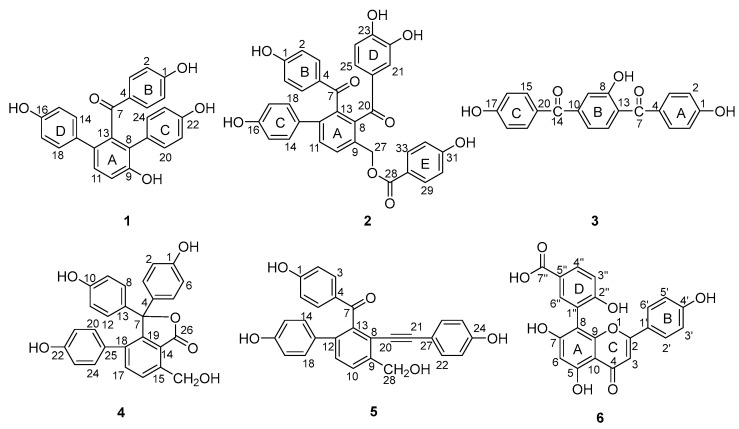
Structures of compounds **1**–**6** isolated from *Selaginella tamariscina*.

The phytochemical study resulted in the isolation of three new benzophenones (**1**–**3**), two known selaginellins and one known flavonoid from *Selaginella tamariscina* (Figure 1). The structures of all new compounds were elucidated through extensive spectroscopic data. The known compounds were identified as selaginellin H (**4**) [30], selaginellin S (**5**) [31], and unciflavone D (**6**) [32] by comparing their NMR data with those reported in the literature.

**Figure 2:**
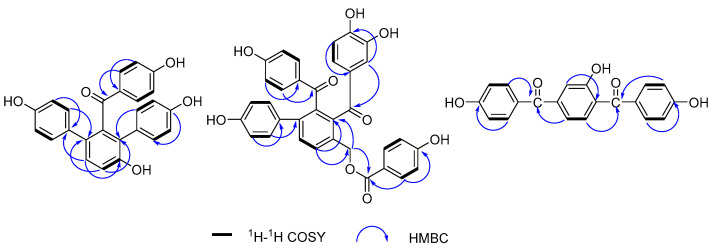
Selected HMBC and ^1^H-^1^H COSY correlations of **1**–**3**.

**Figure 3:**
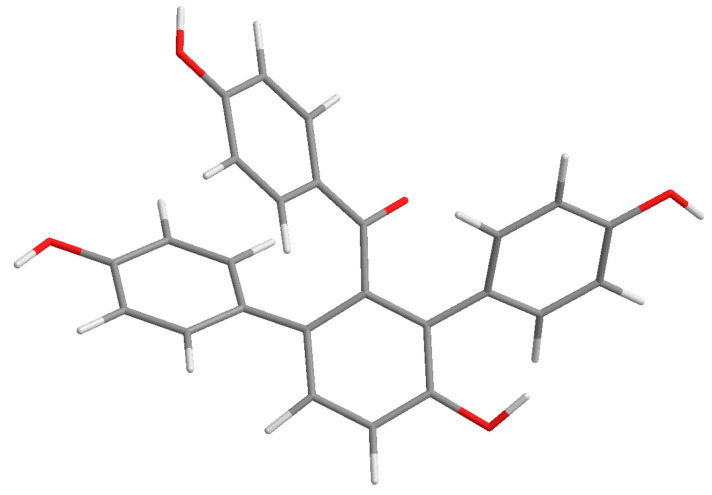
The 3D structure of compound **1**.

Compound **1** was purified as a yellow amorphous powder. The molecular formula was elucidated as C_25_H_18_O_5_ based on its [M + H]^+^ quasi-molecular ion peak at 399.1243 (calcd for C_25_H_19_O_5_, 399.1232) in the HR–ESI–MS, which indicated 17 unsaturations. This molecular formula was consistent with the ^1^H and ^13^C NMR data (Table 1). The ^1^H NMR spectrum of **1** (Figure S1) exhibited signals for three para-substituted phenyls at *δ* 7.36 (2H, d, *J* = 8.7 Hz), 6.59 (2H, d, *J* = 8.7 Hz), *δ* 7.02 (2H, d, *J* = 8.5 Hz), 6.58 (2H, d, *J* = 8.5 Hz), and *δ* 6.96 (2H, d, *J* = 8.2 Hz), 6.60 (2H, d, *J* = 8.2 Hz), and one orthotetra-substituted phenyls at *δ* 7.20 (1H, d, *J* = 8.4 Hz), 7.05 (1H, d, *J* = 8.4 Hz) on the basis of ^1^H-^1^H COSY spectrum. The ^13^C NMR spectrum of **1** (Figure S2) showed 25 carbon resonances including the corresponding 24 aromatic carbon and one carbonyl carbon at *δ* 198.7 (C-7). According to these spectroscopic data compared with our reported selagibenzophenone C [20,21], compound **1** was inferred to be a benzophenone carrying two phenyl groups. The ^1^H-^1^H COSY spectrum confirmed that ring A was an orthotetra-substituted benzene ring. In the HMBC spectrum (Figure 2), the correlations were observed for H-10, 20, 24 to C-8, which indicated ring C was attached at C-8. The correlations for H-10, 14, 18 to C-12 indicated ring D was attached at C-12. The correlations for H-3, 5 to C-7 along with the weak correlation for H-11 to C-7 evidenced the benzophenone nucleus structure. Except for the above signals, the remaining hydroxyl should be located at C-9 because of the HMBC correlations for H-10, 11 to C-9. Therefore, the structure of compound **1** was elucidated and named selagibenzophenone D, and its 3D structure was shown in Figure 3. To our knowledge, compound **1** represents the second example of diarylbenzophenone from natural sources.

**Figure 4:**
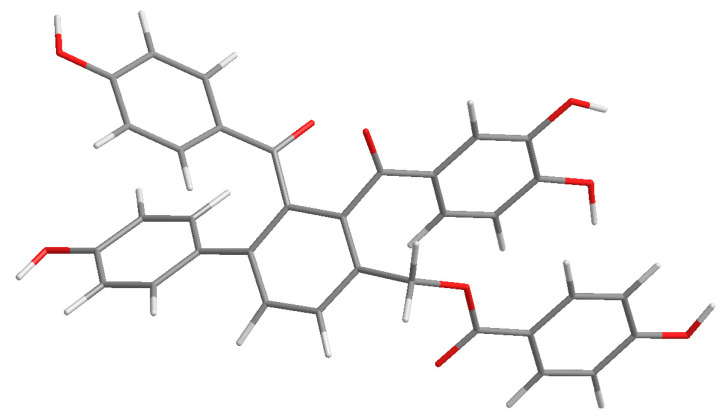
The 3D structure of compound **2**.

Compound **2** was purified as yellow amorphous powder. The molecular formula was deduced as C_34_H_24_O_9_ from its [M + H]^+^ quasi-molecular ion peak at 577.1516 (calcd for C_34_H_25_O_9_, 577.1499) in the HR–ESI–MS spectrum. This molecular formula was consistent with the ^1^H and ^13^C NMR data (Table 1). The ^1^H NMR spectrum of **2** (Figure S7)showed two para-substituted phenyls at *δ* 7.35 (2H, d, *J* = 8.7 Hz), 6.56 (2H, *J* = 8.7 Hz), and *δ* 7.11 (2H, d, *J* = 8.6 Hz), 6.63 (2H, *J* = 8.7 Hz), and one orthotetra-substituted phenyls at *δ* 7.78 (2H, *J* = 7.9 Hz), 7.62 (2H, *J* = 7.9 Hz), which was confirmed by the ^1^H-^1^H COSY spectrum. These above *^1^*H NMR spectral signals of **2** showed some similarity to those of **1** including the signals of rings A, B and C, implying the similar biphenylbenzophenone skeleton. The ^1^H NMR spectrum of **2** exhibited signals of two more benzene rings; one is *p*-hydroxyphenyl (ring E) at *δ* 7.60 (2H, d, *J* = 8.7 Hz) and 6.69 (2H, d, 8.7 Hz), and the other is a *O*-dihydroxyphenyl (ring D) at *δ* 6.84 (1H, dd, *J* = 8.6, 2.2 Hz), 6.66 (2H, m). One oxymethylene signal was also observed at 5.35 (2H, s), confirmed by DEPT experiment. The ^13^C NMR spectrum of **2** (Figure S8) showed 34 carbon resonances including the corresponding 30 aromatic carbon, three carbonyl carbon at *δ* 166.0 (C-28), 196.7 (C-7) and 202.2 (C-20), and one methylene carbon signal at 63.6 (C-27). In the HMBC spectrum (Figure 2), the correlations of H-21, 25 to C-20 and the weak correlations of H-10 to C-20 indicated that ring C and ring A were linked with C-20. The correlations of H-10 to C-13, 27 and H-27 to C-10, C-13 indicated that the methylene was attached to C-9. The correlations of H-29, 33 to C-28, C-31 and H-30, 32 to C-34 confirmed ring E was a *p*-hydroxybenzoyloxy, which was located at C-27 evidenced by the HMBC couplings of H-27 to C-28. Therefore, the structure of compound **2** was elucidated and named selagibenzophenone E, and its 3D structure was shown in Figure 4. Compound **2** possesses an unusual biphenyl-bisbenzophenone structure. It seems that compound **2** and compound **5** (selaginellin S) have a similar substitution pattern in ring A. Selaginellin S belongs to the selaginellin family with the parent nucleus structure of an alkynylphenol. Li et al. reviewed such compounds from the genus of Selaginella and summarized the proposed biosynthetic pathways [1]. These proposed that compound **2** originated from the similar precursor.

**Figure 5:**
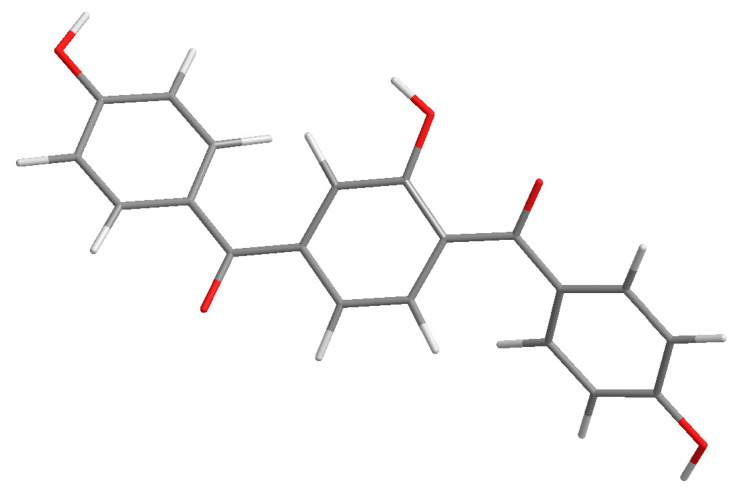
The 3D structure of compound **3**.

Compound **3** was purified as yellow amorphous powder. The molecular formula of C_20_H_14_O_5_ was analyzed from its [M + H]^+^ quasi-molecular ion peak at 335.0918 (calcd for C_20_H_15_O_5_, 335.0919) in the HR–ESI–MS spectrum. The ^1^H NMR spectrum of **3** (Figure S14) exhibited signals for two para-substituted phenyls at *δ* 7.56 (2H, d, *J* = 8.5 Hz), 6.76 (2H, d, *J* = 8.5 Hz) and *δ* 7.59 (2H, d, *J* = 8.5 Hz), 6.78 (2H, d, *J* = 8.5 Hz), and one 1,2,4-trisubstituted phenyls at *δ* 7.53 (1H, d, *J* = 8.5 Hz), 7.01 (1H, dd, *J* = 8.5, 2.2 Hz), 6.92 (1H, d, *J* = 2.2 Hz), which was confirmed by the ^1^H-^1^H COSY spectrum. The ^13^C NMR spectrum of **3** (Figure S15) showed 20 carbon resonances including the corresponding 18 aromatic carbon and two carbonyl carbon at *δ* 195.3 (C-7) and 196.5 (C-14). In HMBC spectrum, the correlations of H-3, 5 to C-7 and H-12 to C-7 indicated that ring A and ring B were connected with C-7. The HMBC couplings of H-9, 11 to C-14 and H-15, 19 to C-14 indicated that ring B and ring C were linked with C-14. In addition, the HMBC couplings of H-9, 12 to C-8 defined the location of a hydroxyl at C-8. Thus, the structure of compound **3** was elucidated and given a successive name, selagibenzophenone F, and its 3D structure was shown in Figure 5.

**Table 1:** ^1^H (400 MHz) and ^13^C NMR (100 MHz) spectral data of **1**–**3** in MeOH-*d*_4_.

	1		2		3	
Position	*δ*_H_ (*J* in Hz)	*δ* *_C_*	*δ*_H_ (*J* in Hz)	*δ* *_C_*	*δ*_H_ (*J* in Hz)	*δ* *_C_*
1	-	162.4	-	162.4	-	162.2
2	6.59 (1H, d, 8.7)	114.2	6.56 (1H, d, 8.7)	114.4	6.76 (1H, d, 8.5)	114.6
3	7.36 (1H, d, 8.7)	132.0	7.35 (1H, d, 8.7)	132.4	7.56 (1H, d, 8.5)	132.2
4	-	131.8	-	128.8	-	128.8
5	7.36 (1H, d, 8.7)	132.0	7.35 (1H, d, 8.7)	132.4	7.56 (1H, d, 8.5)	132.2
6	6.59 (1H, d, 8.7)	114.2	6.56 (1H, d, 8.7)	114.4	6.76 (1H, d, 8.5)	114.6
7	-	198.7	-	196.7	-	195.3
8	-	126.6	-	137.9	-	160.0
9	-	153.2	-	137.4	6.92 (1H, d, 2.2)	115.6
10	7.05 (1H, d, 8.4)	115.7	7.62 (1H, d, 7.9)	130.6	-	143.2
11	7.20 (1H, d, 8.4)	129.4	7.78 (1H, d, 7.9)	130.8	7.01 (1H, dd, 8.5, 2.2)	115.6
12	-	131.4	-	141.0	7.53 (1H, d, 8.5)	132.2
13	-	140.2	-	132.2	-	130.2
14	6.96 (1H, d, 8.2)	131.5	7.11 (1H, d, 8.6)	130.0	-	196.5
15	6.60 (1H, d, 8.2)	114.4	6.63 (1H, d, 8.6)	114.8	7.59 (1H, 8.5)	132.0
16	-	156.0	-	157.0	6.78 (1H, 8.5)	114.6
17	6.60 (1H, d, 8.2)	114.4	6.63 (1H, d, 8.6)	114.8	-	162.5
18	6.96 (1H, d, 8.2)	131.5	7.11 (1H, d, 8.6)	130.0	6.78 (1H, 8.5	114.6
19	-	126.8	-	137.5	7.59 (1H, 8.5)	132.0
20	7.02 (1H, d, 8.5)	130.0	-	202.2	-	129.2
21	6.58 (1H, d, 8.5)	114.0	6.66 (1H, m)	117.8		
22	-	155.9	-	148.9		
23	6.58 (1H, d, 8.5)	114.0	-	155.2		
24	7.02 (1H, d, 8.5)	130.0	6.66 (1H, m)	117.2		
25	-	129.8	6.84 (1H, dd, 8.6, 2.2)	124.9		
26			-	120.2		
27			5.35 (2H, s)	63.6		
28			-	166.0		
29			7.60 (1H, d, 8.7)	131.4		
30			6.69 (1H, d, 8.7)	114.6		
31			-	162.1		
32			6.69 (1H, d, 8.7)	114.6		
33			7.60 (1H, d, 8.7)	131.4		
34			-	120.0		

### 2.2. Cytotoxic Effects against Cancer Cells

Much research has indicated that the phytochemicals from the genus of *Selaginella* exhibited good anticancer effects. Therefore, compounds **1**–**6** were evaluated for their cytotoxity against human hepatocellular carcinoma HepG2 and SMCC-7721 cells (Table 2) in this study. Compound **2** showed moderate inhibitory activity against HepG2 and SMCC-7721 cells with IC_50_ values of 32. 575 and 15.816 µM, respectively. And compounds **4** and **5** exhibited moderate inhibitory activity against HepG2 cells, while no activity against SMCC-7721 cells. Other compounds showed no activity on the two cell lines.

### 2.3. NO Inhibitory Activities

**Figure 6:**
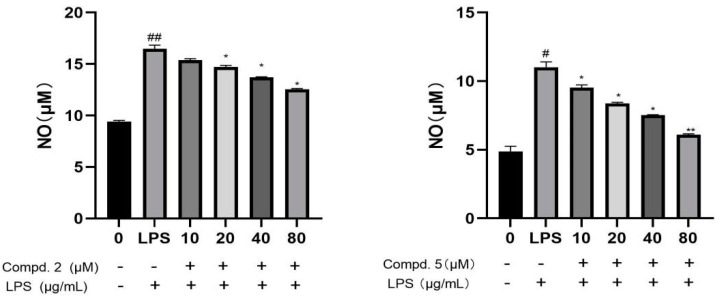
The NO inhibitory activity of compounds **2** and **5** in LPS-activated RAW264.7 cells. #, ## *p* < 0.001 vs. control group, *, ** *p* < 0.001 vs. LPS group (*n* = 3).

NO was considered as a key inflammatory mediator which may be helpful to treat the inflammation. Some research confirmed that such polyphenols showed NO inhibitory activity, so compounds **1**–**6** were assayed for their NO inhibitory effects in RAW 264.7 cells. The experimental results indicated that compounds **2** and **5** showed moderate anti-inflammatory activity by inhibiting the release of NO from RAW264.7 mouse macrophages (Figure 6).

## 3. Materials and Methods

### 3.1. General Experimental Procedures

^1^H NMR, ^13^C NMR, Distortionless Enhancement by Polarization Transfer (DEPT), ^1^H-^1^H Correlated Spectroscopy (^1^H-^1^H COSY), Heteronuclear Multiple Quantum Correlation (HMQC), and Heteronuclear Multiple Bond Correlation (HMBC) experiments were performed on a Bruker Avance 400 MHz NMR spectrometer (Santa Clara, CA, USA) in MeOH-*d*_4_ with TMS as an international standard. High resolution mass spectra were obtained on a Agilent G6500 Series Q-TOF mass spectrometer (ESI-MS) (Agilent Technologies Inc., Santa Clara, CA, USA). Analytic high-performance liquid chromatography (HPLC) was performed using on an Agilent 1200/1260 Series HPLC system (Agilent Technologies Inc., USA) equipped with a four-pump with an in-line degasser, autosampler, oven and Diode-array detector (DAD) with a YMC C18 (5 μm, 4.6 × 250 mm) column. The silica gel (200–300 mesh, Qingdao Marine Chemical Inc., Qingdao, China), HPD-100 Macroporous resin (Beijing Credit Technology Co., Ltd., Beijing, China) and ODS-A (YMC-GEL, YMC Co., Ltd., Kyoto, Japan) were used for open column chromatography (CC). The semi-preparative HPLC was performed on an Agilent 1260 Series HPLC system using a YMC ODS-A chromatographic column (10 μm, 10 × 250 mm). GF254 plates (Qingdao Marine Chemical Inc., Qingdao, China) and reversed-phase silica gel plates (Merck, Darmstadt, Germany) were used for TLC analysis. The fractions were monitored by TLC, and the spots were visualized by heating the silica gel plates after spraying with 5%H_2_SO_4_ in EtOH.

### 3.2. Plant Material

The air-dried whole herbs of *Selaginella tamariscina* were collected from ShaoYang of Hunan Province, China, in October 2017 and identified by Prof. Xi-Feng Sheng (Hunan Normal University, Changsha, China). A voucher specimen (No. JB-2017) had been deposited in the Laboratory of Phytochemistry, School of Medicine, Hunan Normal University.

### 3.3. Extraction and Isolation

The air-dried *S. tamariscina* (30 kg) was extracted with 70% EtOH (10 L × 2 h × 2 times) and filtered. The conbined extract was concentrated to 5 L and suspended in H_2_O (5 L) and partitioned successively with petroleum ether, EtOAc and n-BuOH to yield three portions. The n-BuOH portion was subjected to a macroporous resin (HPD-100) column with EtOH-H_2_O gradient elution (30%, 50%, 70%, 95%) to obtain 4 fractions (A-D). The 50% portion (B) was subjected to a silica gel column (200–300 mesh) eluted with CH_2_Cl_2_/MeOH (from 100:0 to 0:100, *v*/*v*) to obtain one hundred and eighty-six fractions (Fr. B1-B186) based on TLC. Frs. B37-B57 was further separated via ODS column eluted with MeOH-H_2_O (30%, 40%, 50%, 60%, 70%, 80%, 90%, 100%) to provide 134 subfractions (S-Fr. 1–134). The S-Fr. 3 was purified by semi-preparative HPLC (3.0 mL/min, 254 nm) with can–H_2_O (25:75, *v*/*v*) to give compounds **1** (t_R_ = 36.5 min, 6.5 mg), **3** (t_R_ = 21.5 min, 20.0 mg) and **4** (t_R_ = 37.5 min, 12.5 mg). The S-Fr. 11 was respectively purified with semi-preparative HPLC (3.0 mL/min, 254 nm) wicanACN–H_2_O (22:78, *v*/*v*) to give compounds **5** (t_R_ = 55.7 min, 18.0 mg) and **6** (t_R_ = 28.8 min, 7.5 mg). The S-Fr. 35 was further purified with semi-preparative HPLC (3.0 mL/min, 254 nm)canth ACN–H_2_O (38:62, *v*/*v*) to obatain compound **2** (t_R_ = 29.0 min, 12.5 mg).

Selagibenzophenone D (**1**): Yellow powder. UV (MeOH) λ*_max_* (nm; log *ε*): 276 (4.61). ^1^H NMR and ^13^C NMR (MeOH-*d*_4_) see Table 1; HR-ESI-MS calcd for C_25_H_19_O_5_ [M + H]^+^ 399.1243; found 399.1232.

Selagibenzophenone E (**2**): Yellow powder. UV (MeOH) λ*_max_* (nm; log *ε*): 264 (4.26). ^1^H NMR and ^13^C NMR (MeOH-*d*_4_) see Table 1; HR-ESI-MS calcd for C_34_H_25_O_9_ [M + H]^+^ 577.1516; found 577.1499.

Selagibenzophenone F (**3**): Yellow powder. UV (MeOH) λ*_max_* (nm; log *ε*): 290 (4.22). ^1^H NMR and ^13^C NMR (MeOH-*d*_4_) see Table 1; HR-ESI-MS calcd for C_20_H_15_O_5_ [M + H]^+^ 335.0918; found 335.0919.

Selaginellin H (**4**): Light yellow amorphous powder. UV (MeOH) λ_max_ (nm): 226, 264, 334. ^1^H NMR (MeOH-*d*_4_, 400 MHz): *δ*_H_ 7.86 (1H, d, *J* = 8.0 Hz, H-16), 7.58 (1H, d, *J* = 8.0 Hz, H-17), 6.83 (4H, d, *J* = 8.5 Hz, H-3, 5, 8, 12), 6.64 (4H, d, *J* = 8.5 Hz, H-2, 6, 9, 11), 6.51 (2H, d, *J* = 8.5 Hz, H-20, 24), 6.47 (2H, d, *J* = 8.5 Hz, H-21, 23), 5.20 (2H, s, H-26). ^13^C NMR (MeOH-*d*_4_, 100 MHz): *δ*_C_ 170.6 (C-27), 157.4 (C-1, 10), 156.6 (C-22), 151.1 (C-19), 141.5 (C-15), 138.1 (C-18), 137.5 (C-17), 130.5 (C-4, 20, 24), 130.2 (C-3, 5), 129.9 (C-8, 12), 129.8 (C-13), 129.5 (C-16), 129.4 (C-25), 122.0 (C-14), 114.1 (C-2, 6, 9, 11, 21, 23), 93.7 (C-7), 59.3 (C-26).

Selaginellin S (**5**): Yellow powder. UV (MeOH) λ_max_ (nm): 280. ^1^H NMR (MeOH-*d*_4_, 400 MHz): *δ*_H_ 7.70 (1H, d, *J* = 8.0 Hz, H-10), 7.64 (2H, d, *J* = 8.5 Hz, H-3, 5), 7.42 (1H, d, *J* = 8.0 Hz, H-11), 7.11 (2H, d, *J* = 8.0 Hz, H-14, 18), 6.98 (2H, dd, *J* = 7.5, 2.5 Hz, H-22, 26), 6.78 (2H, d, *J* = 8.5 Hz, H-2, 6), 6.67 (2H, dd, *J* = 7.5, 2.5 Hz, H-23, 25), 6.66 (1H, d, *J* = 8.0 Hz, H-15, 17), 4.89 (2H, s, H-28). ^13^C NMR (MeOH-*d*_4_, 100 MHz): *δ*_C_ 197.9 (C-7), 162.9 (C-1), 158.0 (C-24), 156.7 (C-16), 141.3 (C-9), 141.0 (C-13), 138.9 (C-12), 132.4 (C-22, 26), 132.2 (C-3, 5), 130.8 (C-19), 129.8 (C-14, 18), 129.3 (C-11), 129.1 (C-4), 126.8 (C-10), 119.0 (C-8), 115.0 (C-2, 6), 114.9 (C-23, 25), 114.6 (C-15, 17), 113.2 (C-27), 99.2 (C-21), 82.5 (C-20), 61.7 (C-28).

Unciflavone D (**6**): Light yellow amorphous powder. UV (MeOH) λ_max_ (nm): 226, 264, 324. ^1^H NMR (MeOH-*d*_4_, 400 MHz): *δ*_H_ 8.00 (1H, dd, *J* = 8.5, 2.5 Hz, H-4″), 7.98 (1H, d, *J* = 2.5 Hz, H-6″), 7.54 (2H, d, *J* = 8.5 Hz, H-2′, 6′), 7.02 (1H, d, *J* = 8.5 Hz, H-3″), 6.77 (2H, d, *J* = 8.5 Hz, H-3′, 5′), 6.62 (2H, s, H-3), 6.37 (1H, s, H-6). ^13^C NMR (MeOH-*d*_4_, 100 MHz): *δ*_C_ 182.9 (C-4), 165.6 (C-7″), 164.7 (C-2), 162.7 (C-7), 161.2 (C-2″), 160.8 (C-5), 156.1 (C-1″), 159.1 (C-9), 134.8 (C-6″), 130.8 (C-4″), 128.0 (C-2′, 6′), 121.9 (C-5″), 121.8 (C-1′), 118.8 (C-4′), 115.4 (C-3′, 5′), 114.8 (C-3″), 104.9 (C-8), 103.9 (C-10), 101.8 (C-3), 98.8 (C-6).

### 3.4. Cytotoxicity Assay

The human hepatocellular carcinoma HepG2 and SMCC-7721 cell line was purchased from the Cell Resource Center, Shanghai Institutes for Biological Sciences of Chinese Academy of Sciences. The cytotoxicity assay was carried out using the Cell Counting Kit-8 (CCK-8) method. HepG2 and the SMCC-7721 cell line were cultured in DMEM at 37 °C, 5% CO_2_. The cells of the logarithmic growth phase were seeded into 96-well plates with a density of 4000 cells/well in a 200 µL medium, respectively. The cells were treated with all tested compounds at various concentrations (0, 5, 10, 20, 40 and 80 µM), with sorafenib as a positive control. Three parallel holes were located and then incubated for 48 h. Subsequently, the 96 well plate was taken out and 10 µL of CCK-8 was added in DMEM 0.1 mL; meanwhile, two separate holes were used as a blank control, with only 10 µL CCK-8 in DMEM 0.1 mL added to each blank hole. Then it was incubated under the same conditions for 4 h. The optical density (OD) was measured at 450 nm using a Biotek Synergy (Bio-Tek Company, Winooski, VT, USA). The experiment was repeated 3 times. The IC_50_ values were calculated to assure the impact of the drugs on cell growth inhibition rate.

### 3.5. Bioassay for NO Inhibitory Activities

NO represents an important inflammatory factor. In this study, we examined the NO inhibitory effects of compounds **1**–**6** by inhibiting NO release in LPS-induced murine macrophage RAW 264.7 cells. The RAW 264.7 cell line was purchased from the Cell Resource Center, Shanghai Institutes for Biological Sciences of Chinese Academy of Sciences. The cells were cultured in an incubator and DMEM at 37 °C in 5% CO_2_. The cells were seeded in 24-well culture plates (10,000 cells/well) and allowed to adhere for 24 h at 37 °C. A blank control group and a drug group were cultured for 2 h in an incubator. 10 μg/mL of LPS (Sigma-Aldrich, Shanghai, China) per well was added to induce inflammation, and was cultured in the incubator for 24 h. As a parameter of NO synthesis, the nitrite concentration was measured by the Griess reaction using the supernatant of the RAW 264.7 cells. The absorbance was read with a microplate reader (Bio-Tek Company, Winooski, VT, USA) at 540 nm. The experiment was performed three times. SPSS 16.0 and GraphPad Prism 6.01 software were used for statistical analysis.

## 4. Conclusions

In this study, three new benzophenone derivatives, selagibenzophenones D-F (**1**–**3**), two known selaginellins and one known flavonoid were isolated from *Selaginella tamariscina*. The structures of the new compounds were elucidated by spectroscopic analysis. Compound **1** represents the second example of a diarylbenzophenone from natural sources. Compound **2** possesses an unusual biphenyl-bisbenzophenone structure. Compounds **1**–**6** from this plant were evaluated for their cytotoxicity against human hepatocellular carcinoma HepG2 cells and human SMCC-7721 cells. Compound **2** showed moderate inhibitory activity against HepG2 and SMCC-7721 cells with IC_50_ values of 32. 575 and 15.816 µM, respectively. Compounds **4** and **5** exhibited moderate inhibitory activity against HepG2 cells. In addition, all compounds were evaluated for their inhibitory activities on lipopolysaccharide-induced nitric oxide (NO) production in RAW264.7 cells. Compounds **2** and **5** exhibited inhibitory activities on lipopolysaccharide-induced nitric oxide (NO) production.

## Figures and Tables

**Table 2:** The IC_50_ values of compounds **1**–**6** against HepG2 and SMCC-7721 cells.

Compounds	IC_50_ (μM)	
	HepG2	SMCC-7721
1	>80	>80
2	32.575	15.816
3	>80	>80
4	40.928	>80
5	61.521	>80
6	>80	>80
sorafenib	4.796	2.089

## Data Availability

All data presented in this research are available in the article.

## Supplementary Materials

The following supporting information can be downloaded at: https://www.mdpi.com/article/10.3390/molecules28124582/s1, Figure S1: ^1^H NMR spectrum of **1** in MeOH-*d*_4_ (400 MHz); Figure S2: ^13^C NMR spectrum of **1** in MeOH-*d*_4_ (100 MHz); Figure S3: ^1^H-^1^H COSY spectrum of **1** in MeOH-*d*_4_; Figure S4: HSQC spectrum of **1** in MeOH-*d*_4_; Figure S5: HMBC spectrum of **1** in MeOH-*d*_4_; Figure S6: HR-ESI-MS spectrum of **1**; Figure S7: ^1^H NMR spectrum of **2** in MeOH-*d*_4_ (400 MHz); Figure S8: ^13^C NMR spectrum of **2** in MeOH-*d*_4_ (100 MHz); Figure S9: DEPT spectrum of **2** in MeOH-*d*_4_ (100 MHz); Figure S10: ^1^H-^1^H COSY spectrum of **2** in MeOH-*d*_4_; Figure S11: HSQC spectrum of **2** in MeOH-*d*_4_; Figure S12: HMBC spectrum of **2** in MeOH-*d*_4_; Figure S13: HR-ESI-MS spectrum of **2**; Figure S14: ^1^H NMR spectrum of **3** in MeOH-*d*_4_ (400 MHz); Figure S15: ^13^C NMR spectrum of **3** in MeOH-*d*_4_ (100 MHz); Figure S16: ^1^H-^1^H COSY spectrum of **3** in MeOH-*d*_4_; Figure S17: HSQC spectrum of **3** in MeOH-*d*_4_; Figure S18: HMBC spectrum of **3** in MeOH-*d*_4_; Figure S19: HR-ESI-MS spectrum of **3**. Figure S20: ^1^H NMR spectrum of **4** in MeOH-*d*_4_ (400 MHz); Figure S21: ^13^C NMR spectrum of **1** in MeOH-*d*_4_ (100 MHz), Figure S22: ^1^H NMR spectrum of **5** in MeOH-*d*_4_ (400 MHz); Figure S23: ^13^C NMR spectrum of **1** in MeOH-*d*_4_ (100 MHz); Figure S24: ^1^H NMR spectrum of **6** in MeOH-*d*_4_ (400 MHz); Figure S25: ^13^C NMR spectrum of **1** in MeOH-*d*_4_ (100 MHz).

## Sample Availability

Samples of the compounds are not available from the authors.
