# Supplementary material for: Three New Benzophenone Derivatives from Selaginella tamariscina
Source: Molecules. 2023 Jun 6;28(12):4582. doi: 10.3390/molecules28124582 (PMC10304954; doi:10.3390/molecules28124582)
Supplement: Supplementary file 1 [file molecules-28-04582-s001.zip › molecules-2429724-supplementary.pdf]

molecules

Supplementary Material

Three New Benzophenone Derivatives from *Selaginella tamariscina*

Jiayin Long [1,†], Qingqing Mao [1,†], Yujie Peng [1], Lei Liu [1], Yin Hong [1], Honglin Xiang [1], Ming Ma [2], Hui Zou [1,*] and Junwei Kuang [2,*]

[1] Key Laboratory of Study and Discovery of Small Targeted Molecules of Hunan Province, School of Medicine, Hunan Normal University, Changsha 410013, China; 202020191549@hunnu.edu.cn (J.L.); 202020191550@hunnu.edu.cn (Q.M.); 202130192065@hunnu.edu.cn (Y.P.); 201930192015@smail.hunnu.edu.cn (L.L.); 01930192016@hunnu.edu.cn (Y.H.); 13828@hunnu.edu.cn (H.X.)

[2] Key Laboratory of Phytochemical R&D of Hunan Province, Key Laboratory of Chemical Biology & Traditional Chinese Medicine Research of Ministry of Education, College of Chemistry and Chemical Engineering, Hunan Normal University, Changsha 410081, China; mingma@hunnu.edu.cn

* Correspondence: zouhui@hunnu.edu.cn (H.Z.); willkuang@hunnu.edu.cn (J.K.)

† These authors contributed equally to this work.

Tables of Contents	page
**Figure S1:** ^{1}H NMR spectrum of **1** in MeOH-d_4 (400 MHz)	3
**Figure S2:** ^{13}C NMR spectrum of **1** in MeOH-d_4 (100 MHz)	4
**Figure S3:** ^{1}H-^{1}H COSY spectrum of **1** in MeOH-d_4	5
**Figure S4:** HSQC spectrum of **1** in MeOH-d_4	6
**Figure S5:** HMBC spectrum of **1** in MeOH-d_4	7
**Figure S6:** HR-ESI-MS sepctrum of **1**	8
**Figure S7:** ^{1}H NMR spectrum of **2** in MeOH-d_4 (400 MHz)	9
**Figure S8:** ^{13}C NMR spectrum of **2** in MeOH-d_4 (100 MHz)	10
**Figure S9:** DEPT spectrum of **2** in MeOH-d_4 (100 MHz)	11
**Figure S10:** ^{1}H-^{1}H COSY spectrum of **2** in MeOH-d_4	12
**Figure S11:** HSQC spectrum of **2** in MeOH-d_4	13
**Figure S12:** HMBC spectrum of **2** in MeOH-d_4	14
**Figure S13:** HR-ESI-MS sepctrum of **2**	15
**Figure S14:** ^{1}H NMR spectrum of **3** in MeOH-d_4 (400 MHz)	16
**Figure S15:** ^{13}C NMR spectrum of **3** in MeOH-d_4 (100 MHz)	17
**Figure S16:** ^{1}H-^{1}H COSY spectrum of **3** in MeOH-d_4	18
**Figure S17:** HSQC spectrum of **3** in MeOH-d_4	19
**Figure S18:** HMBC spectrum of **3** in MeOH-d_4	20
**Figure S19:** HR-ESI-MS sepctrum of **3**	21
**Figure S20:** ^{1}H NMR spectrum of **4** in MeOH-d_4 (400 MHz)	22
**Figure S21:** ^{13}C NMR spectrum of **4** in MeOH-d_4 (100 MHz)	23
**Figure S22:** ^{1}H NMR spectrum of **5** in MeOH-d_4 (400 MHz)	24
**Figure S23:** ^{13}C NMR spectrum of **5** in MeOH-d_4 (100 MHz)	25
**Figure S24:** ^{1}H NMR spectrum of **6** in MeOH-d_4 (400 MHz)	26
**Figure S25:** ^{13}C NMR spectrum of **6** in MeOH-d_4 (100 MHz)	27

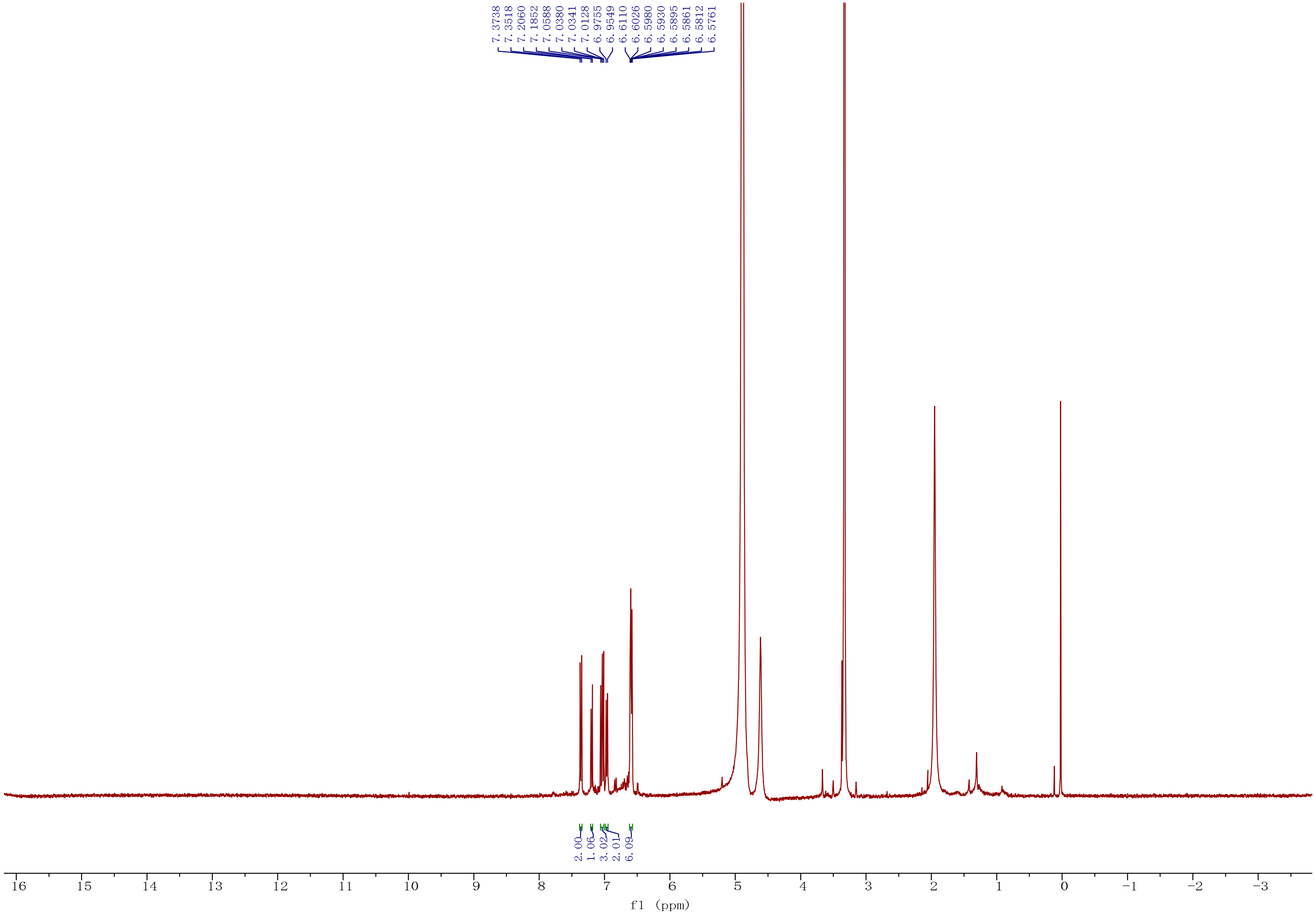

Figure S1: ^{1}H NMR spectrum of **1** in MeOH-d_4 (400 MHz)

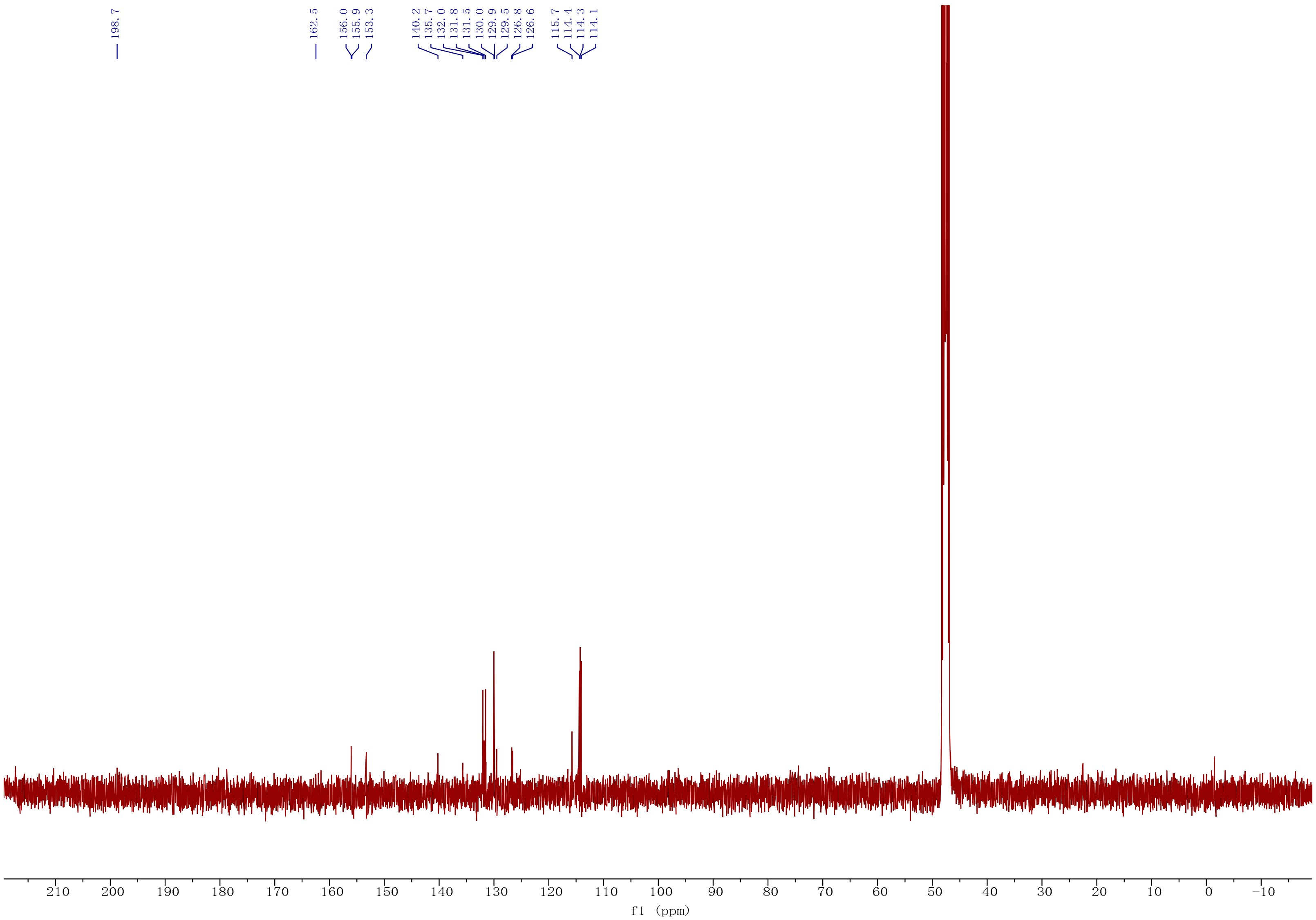

Figure S2: ^{13}C NMR spectrum of **1** in MeOH-d_4 (100 MHz)

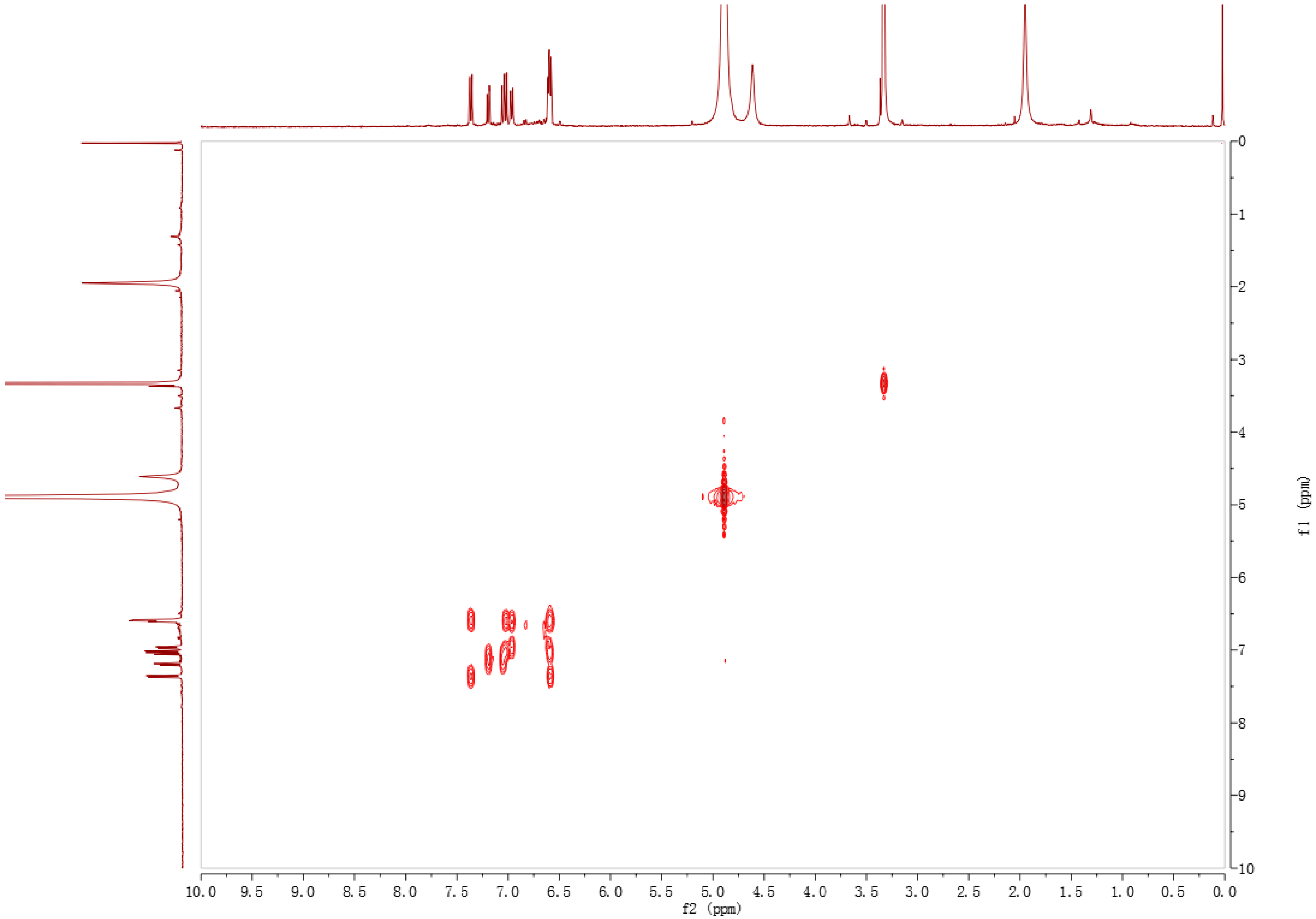

Figure S3: ^{1}H-^{1}H COSY spectrum of **1** in MeOH-*d*$_4$

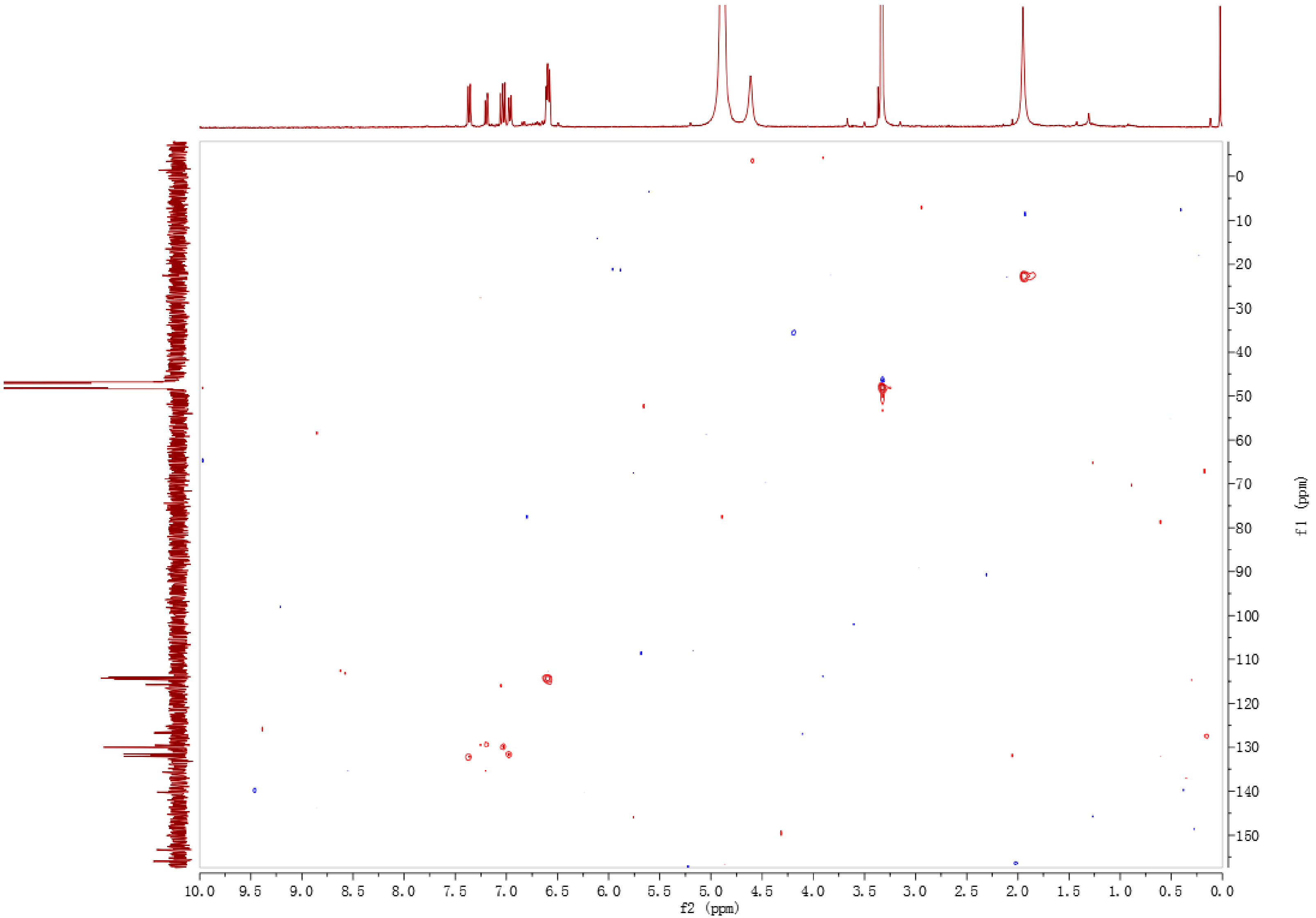

Figure S4: HSQC spectrum of compound **1** in MeOH-d_4

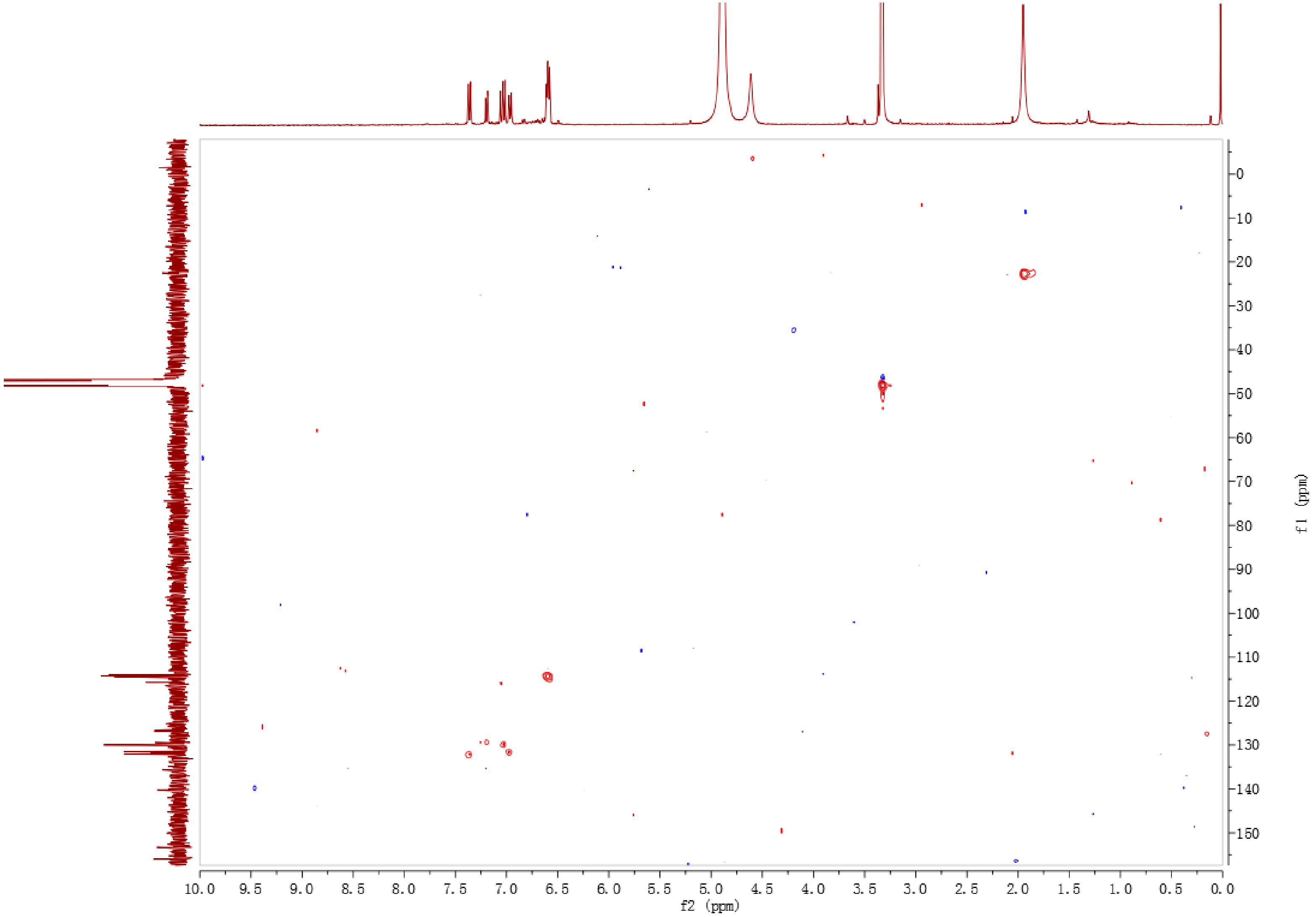

Figure S5: HMBC spectrum of **1** in MeOH-*d*4

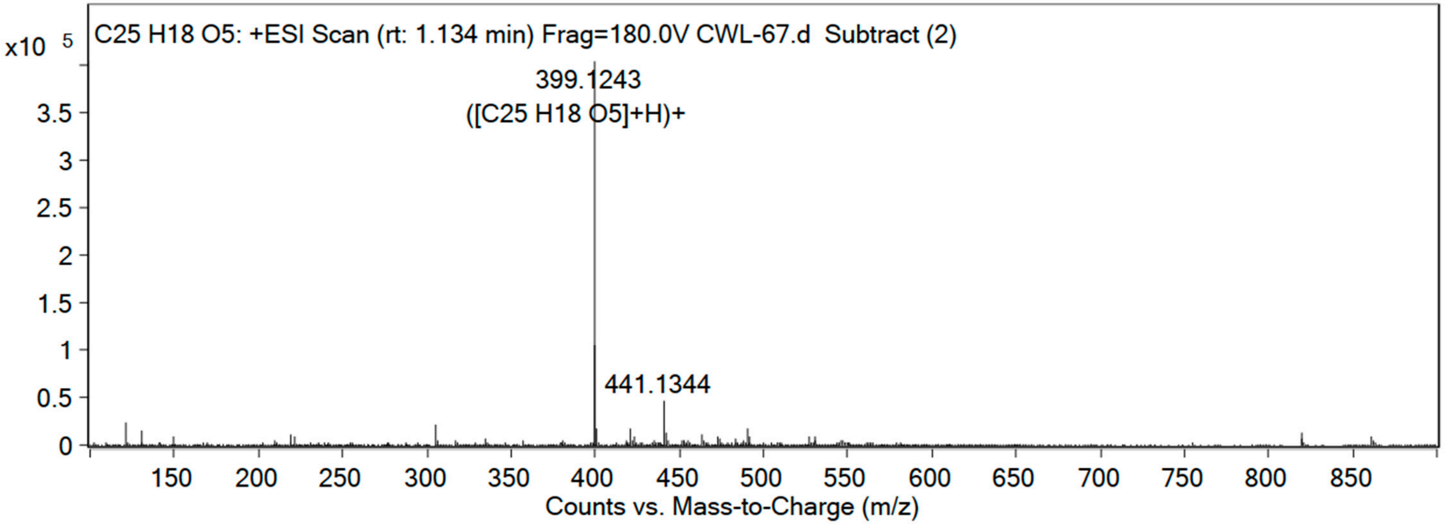

Figure S6. HR-ESI-MS spectrum of **1**.

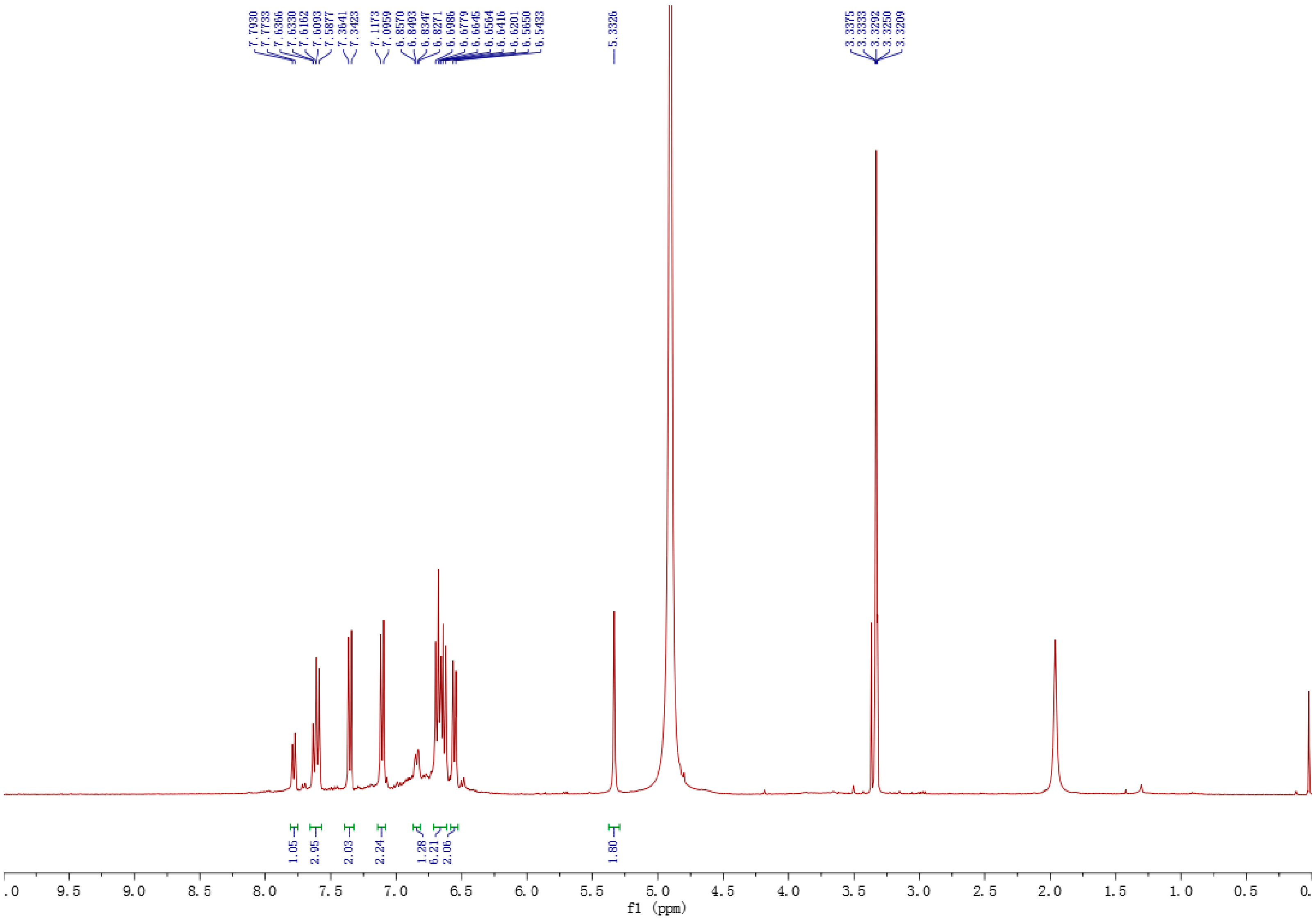

Figure S7: ^{1}H NMR spectrum of **2** in MeOH-d_4 (400 MHz).

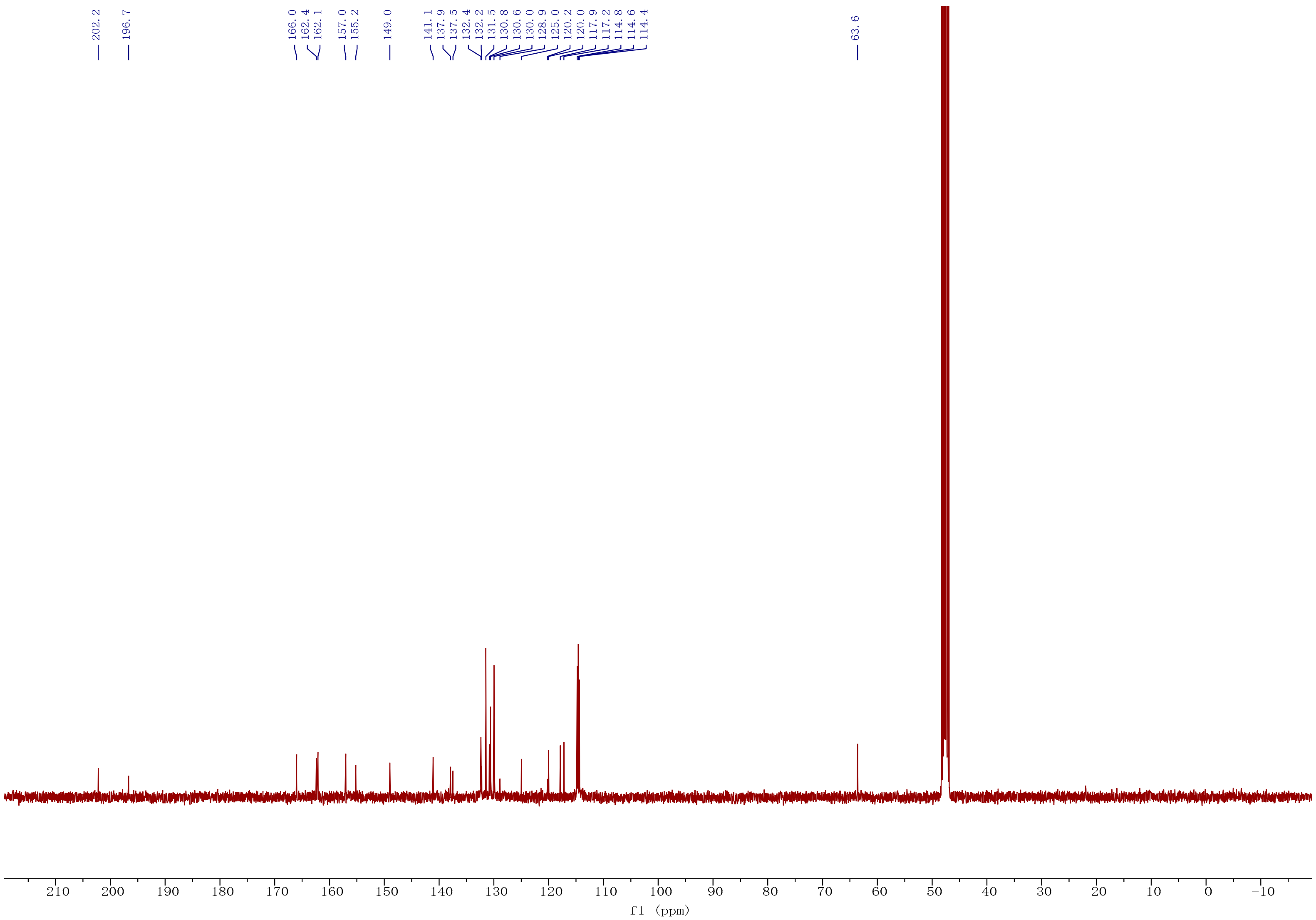

Figure S8: ^{13}C NMR spectrum of **2** in MeOH-d_4 (100 MHz)

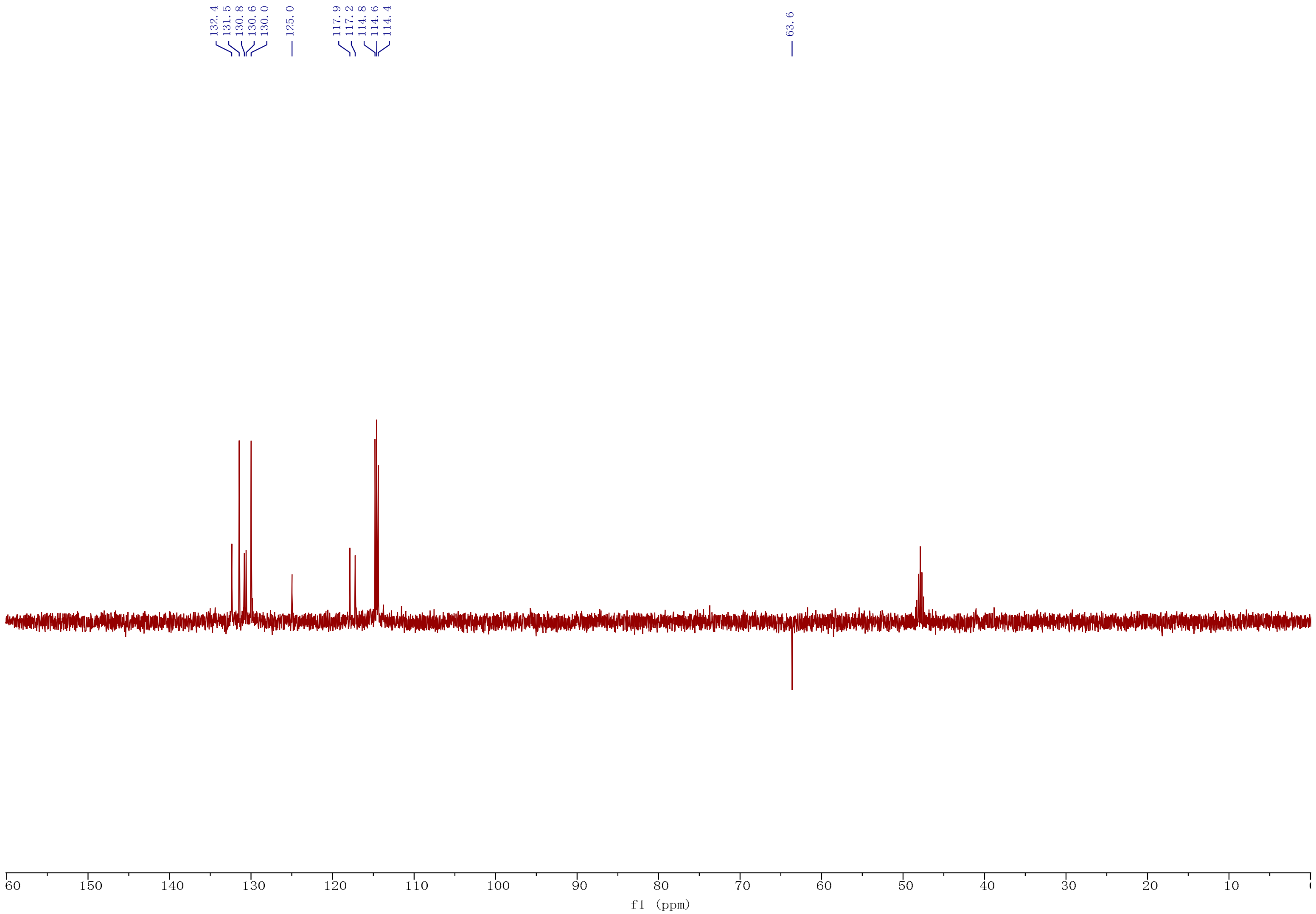

Figure S9: DEPT spectrum of **2** in MeOH-d_4 (100 MHz)

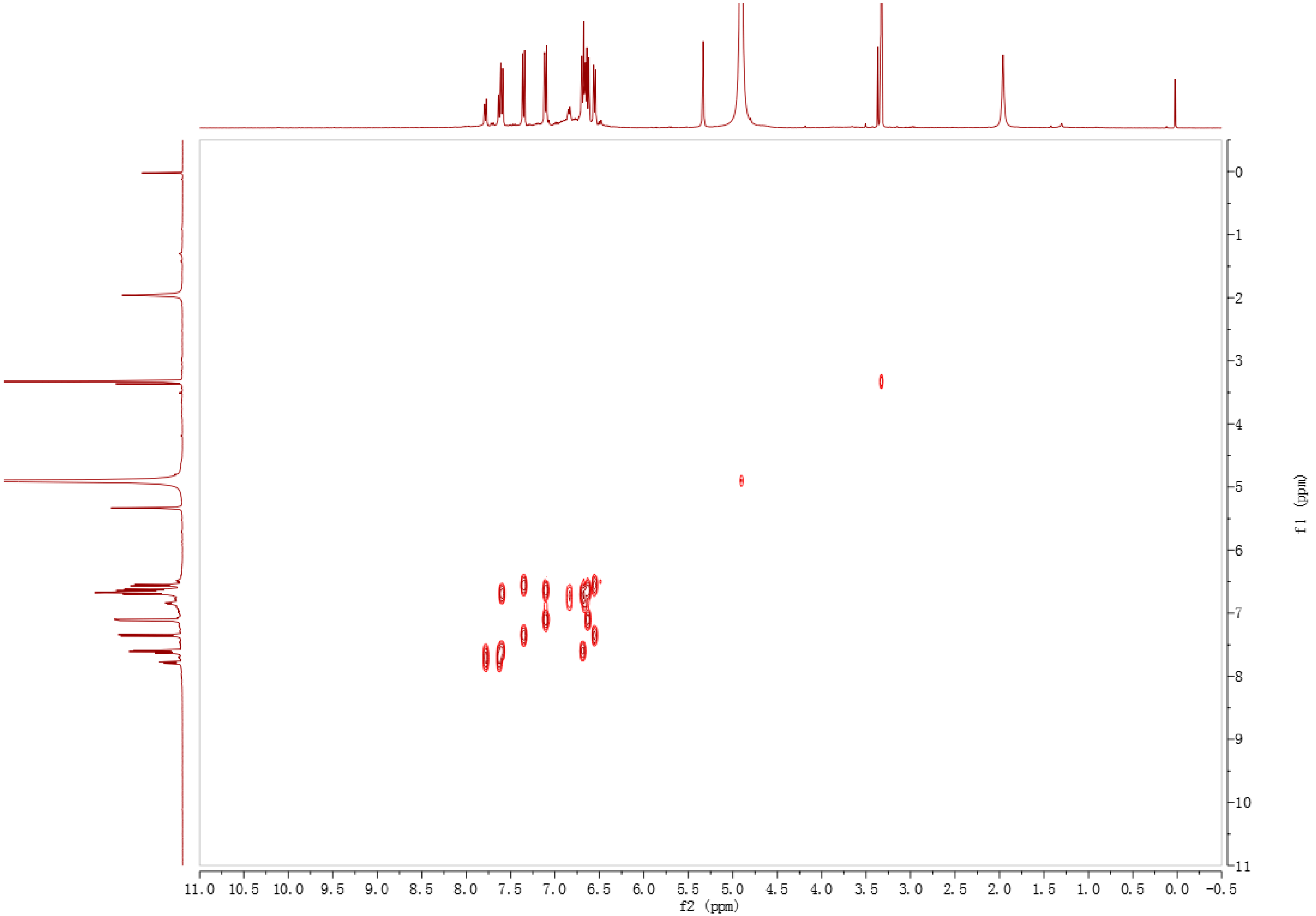

Figure S10: ^{1}H-^{1}H COSY spectrum of **2** in MeOH-d_4

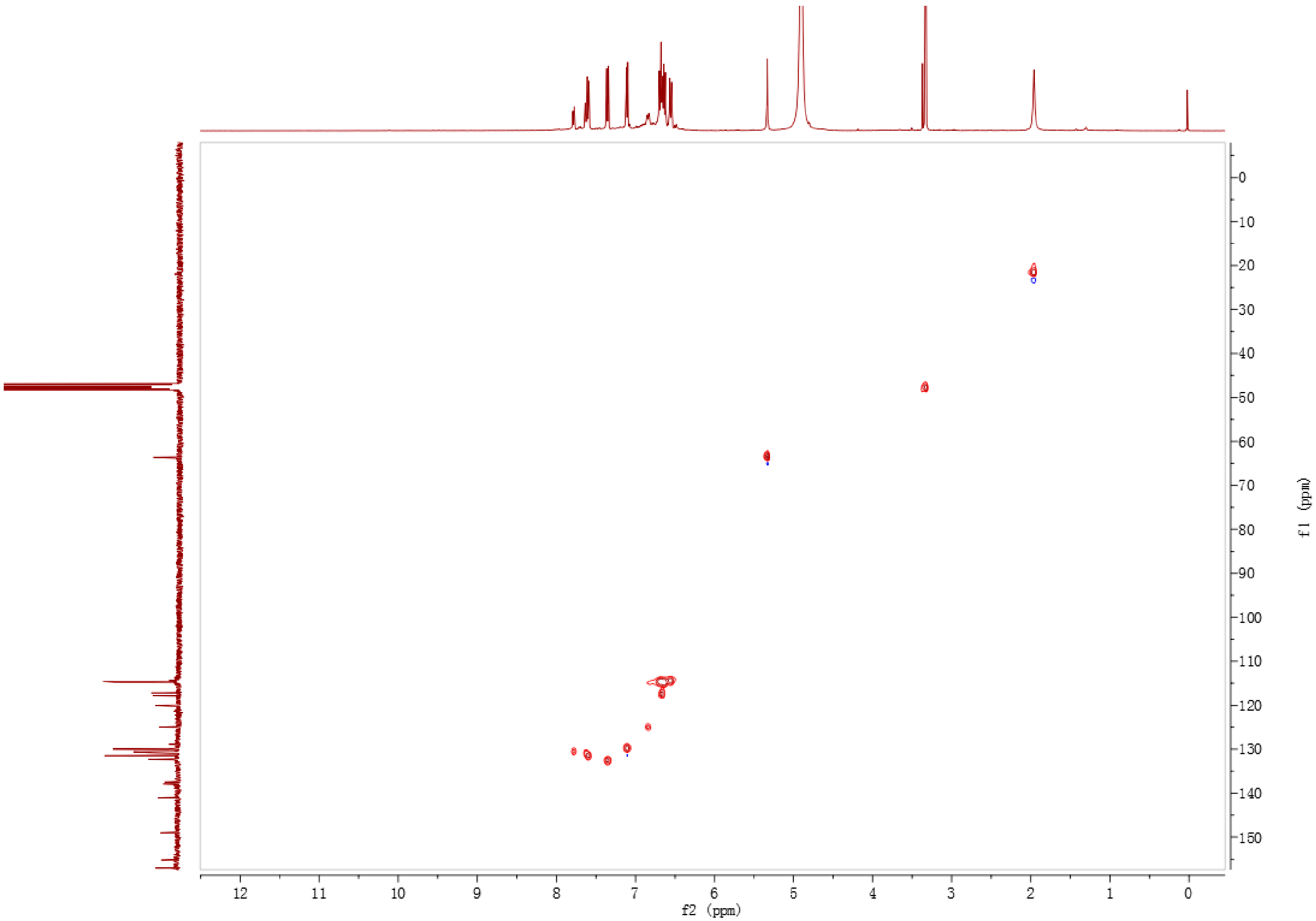

Figure S11: HSQC spectrum of compound **2** in MeOH-d_4

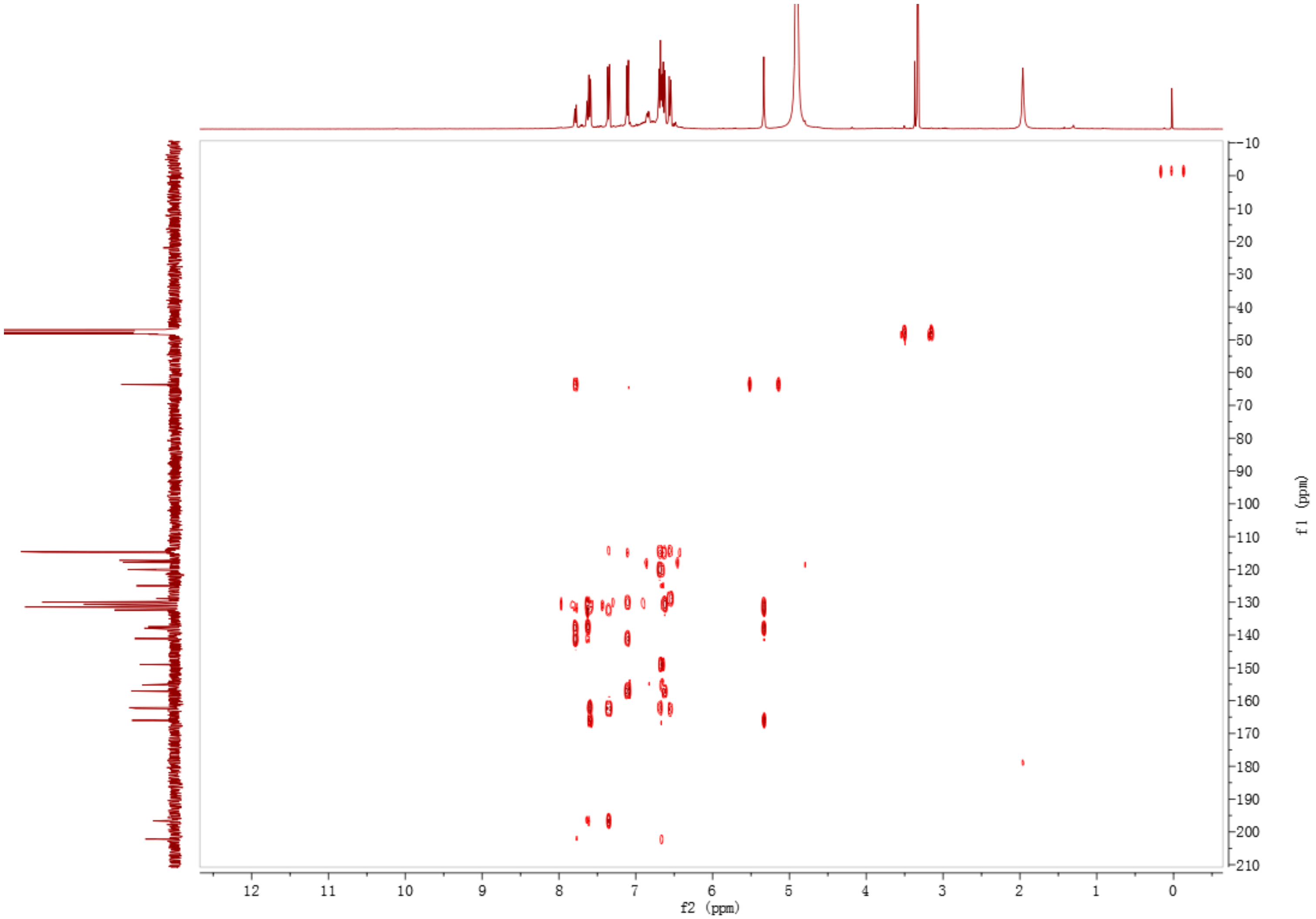

Figure S12: HMBC spectrum of compound **2** in MeOH-d_4

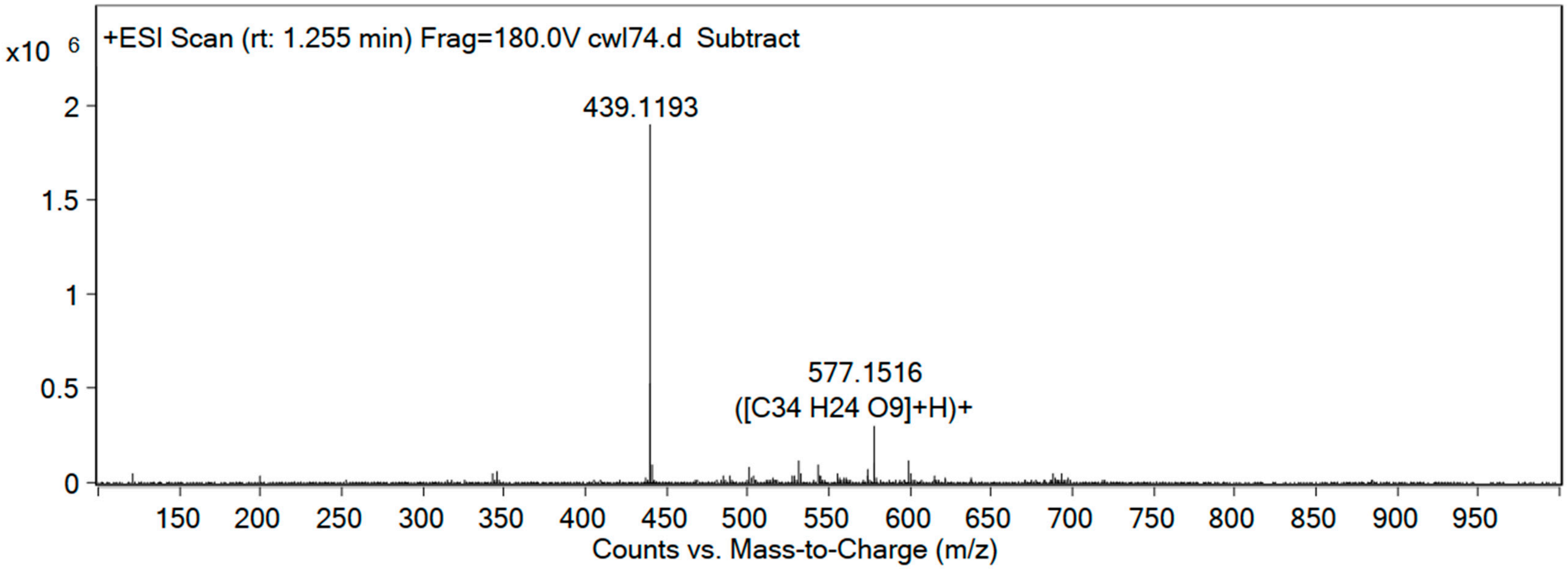

Figure S13: HR-ESI-MS spectrum of **2**

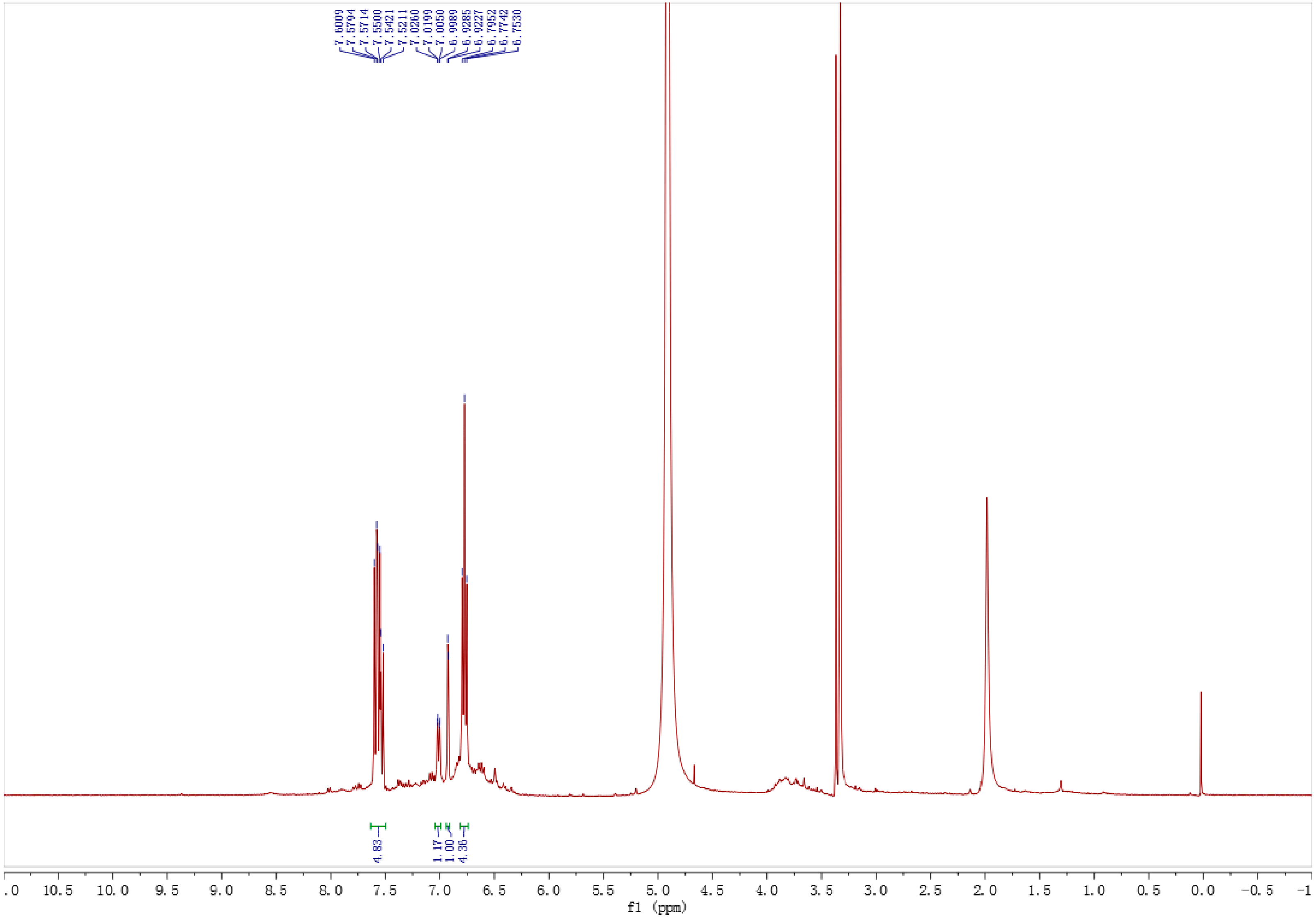

Figure S14: ^{1}H NMR spectrum of **3** in MeOH-d_4 (400 MHz).

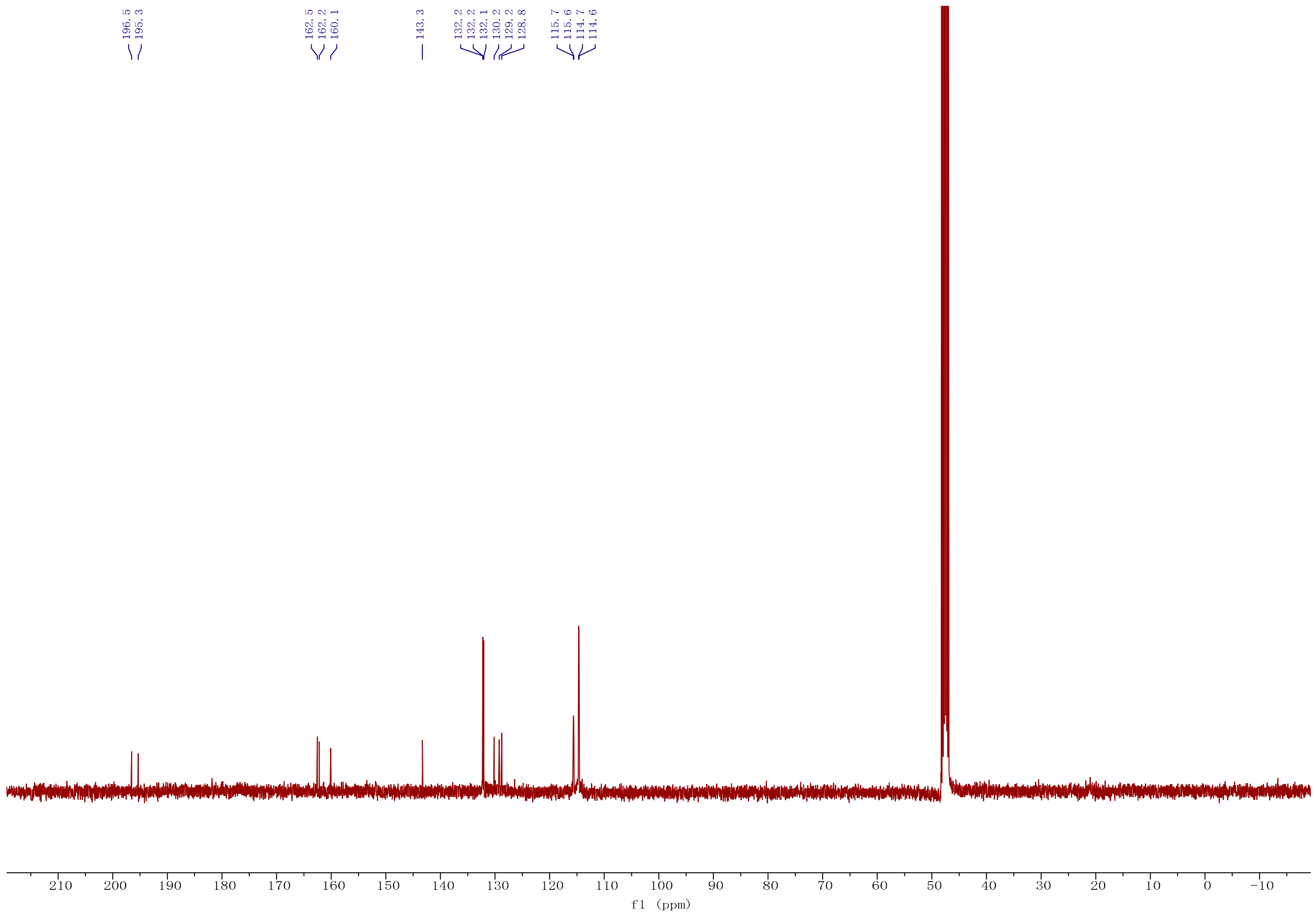

Figure S15: ^{13}C NMR spectrum of **3** in MeOH-d_4 (100 MHz).

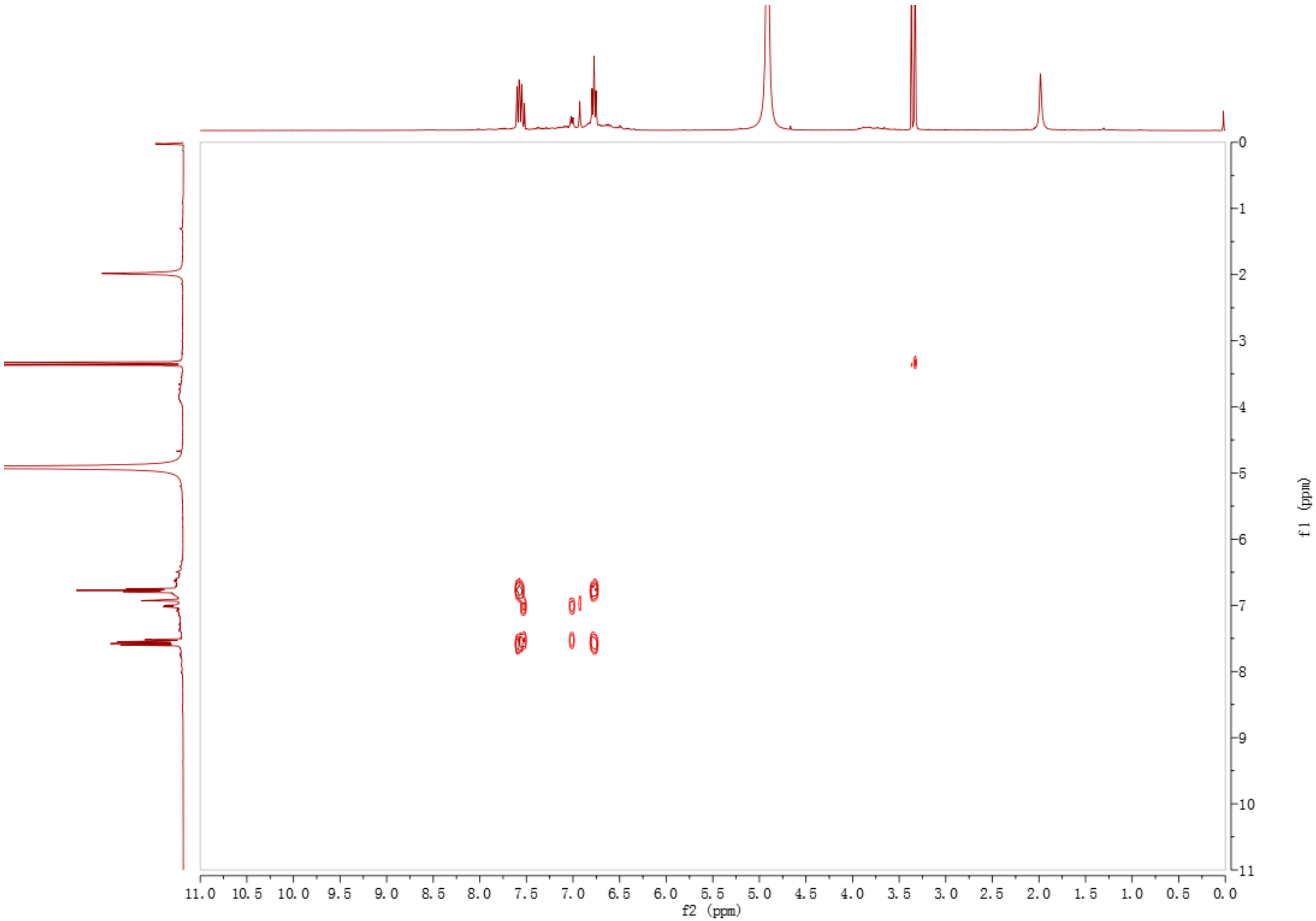

Figure S16: ^{1}H-^{1}H COSY spectrum of **3** in MeOH-d_4.

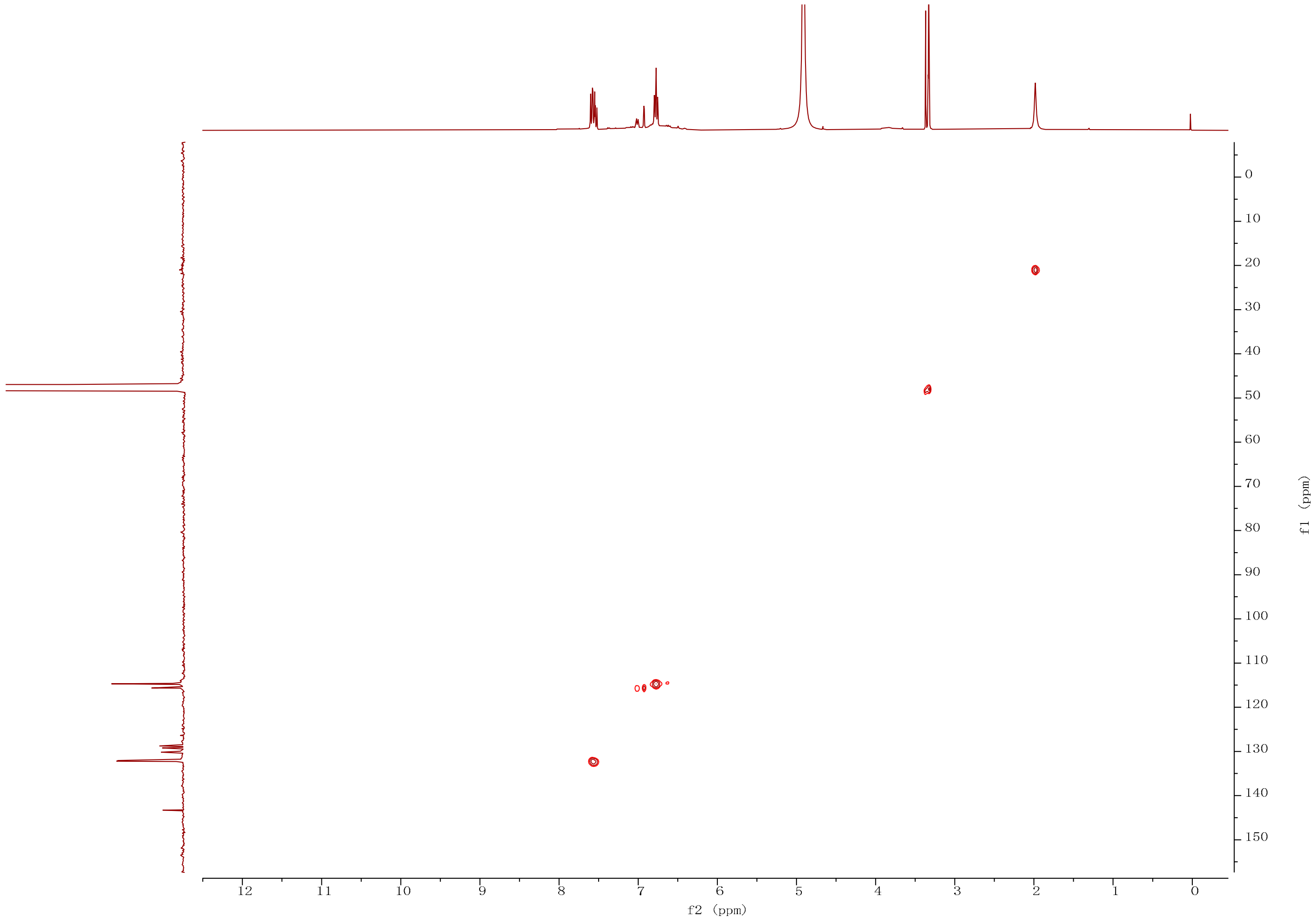

Figure S17: HSQC spectrum of compound **3** in MeOH-*d*4.

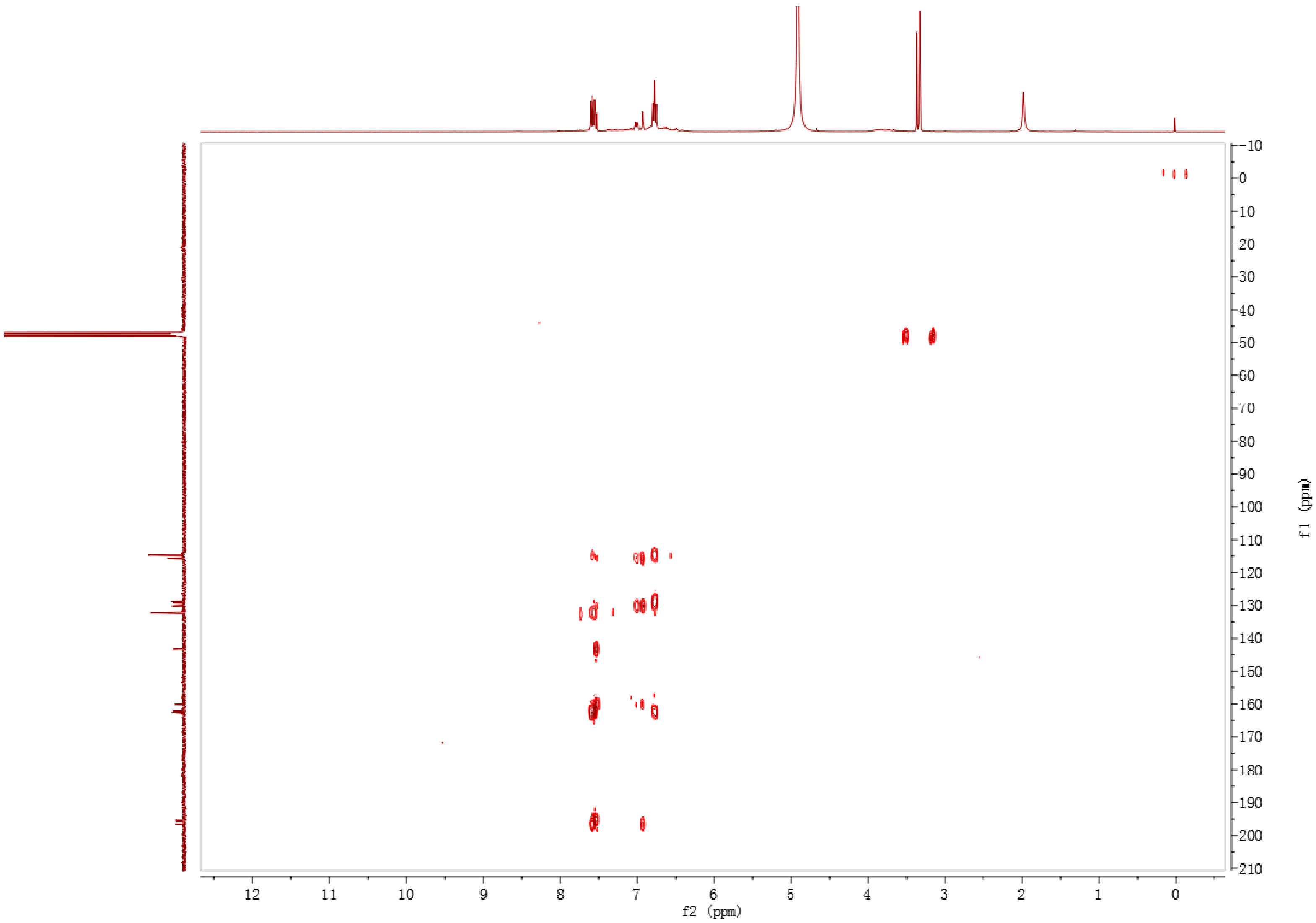

Figure S18: HMBC spectrum of compound **3** in MeOH-d_4.

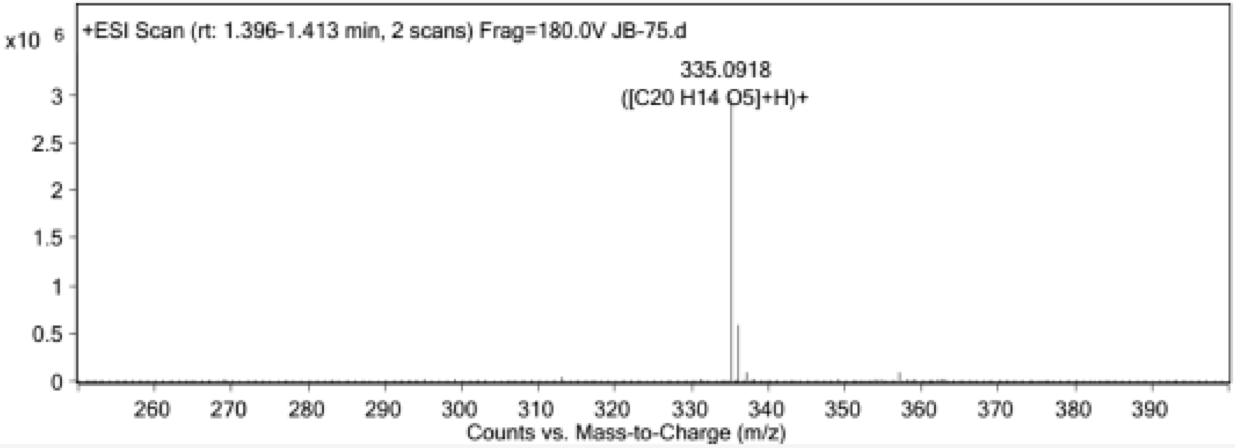

Figure S19: HR-ESI-MS spectrum of **3.**

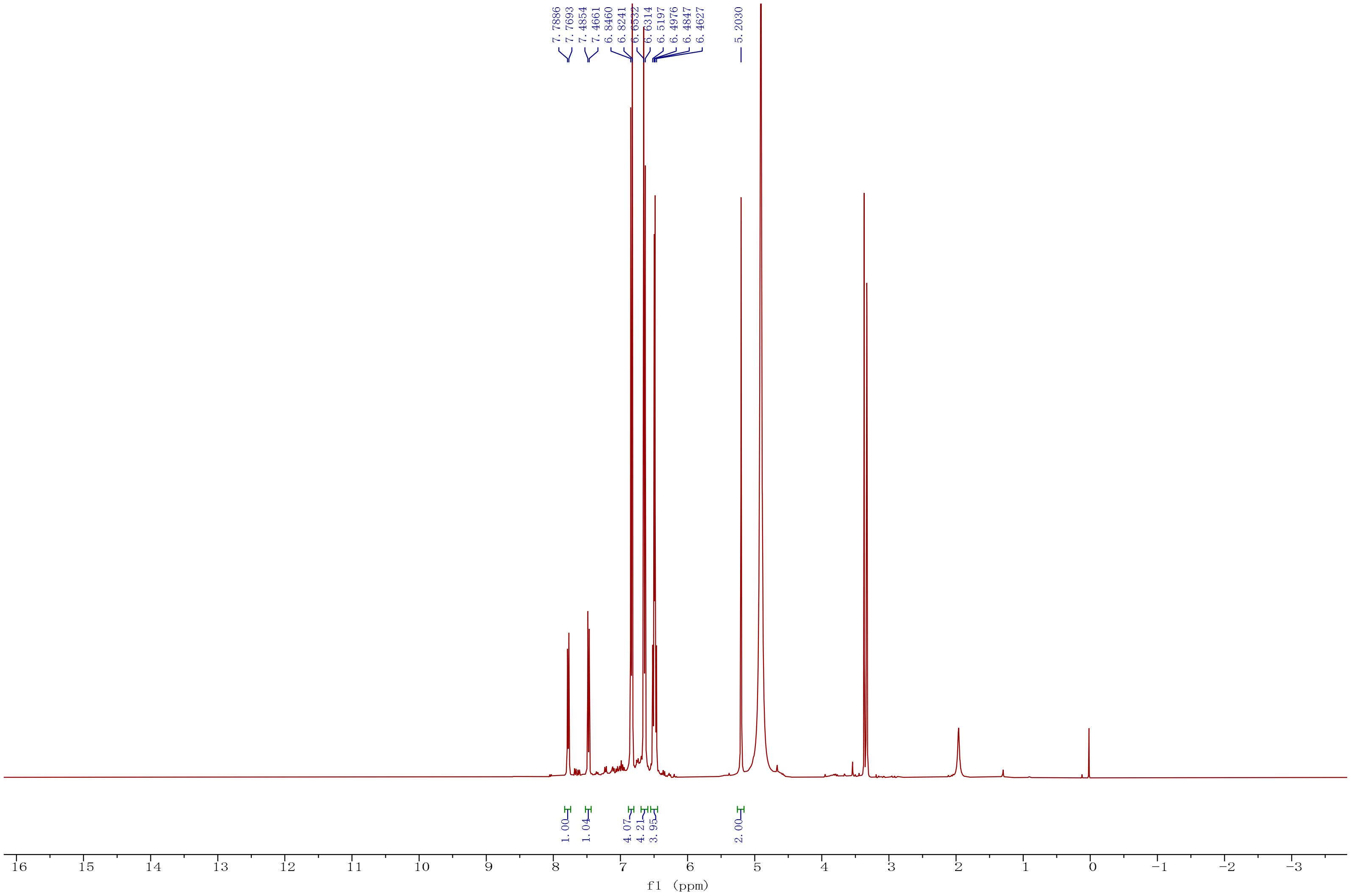

Figure S20: ^{1}H NMR spectrum of **4** in MeOH-d_4 (400 MHz).

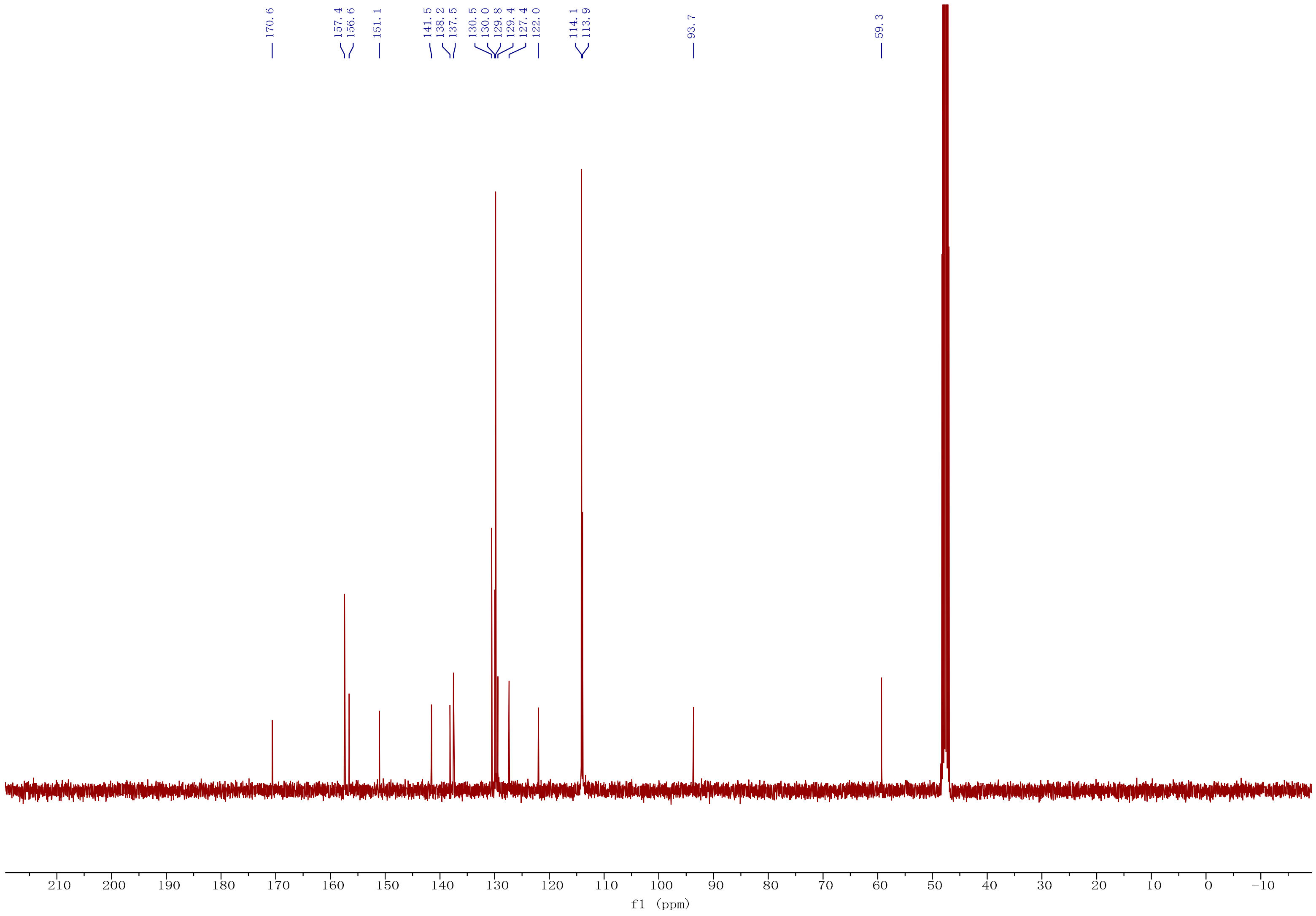

Figure S21: ^{13}C NMR spectrum of **4** in MeOH-*d*4 (100 MHz).

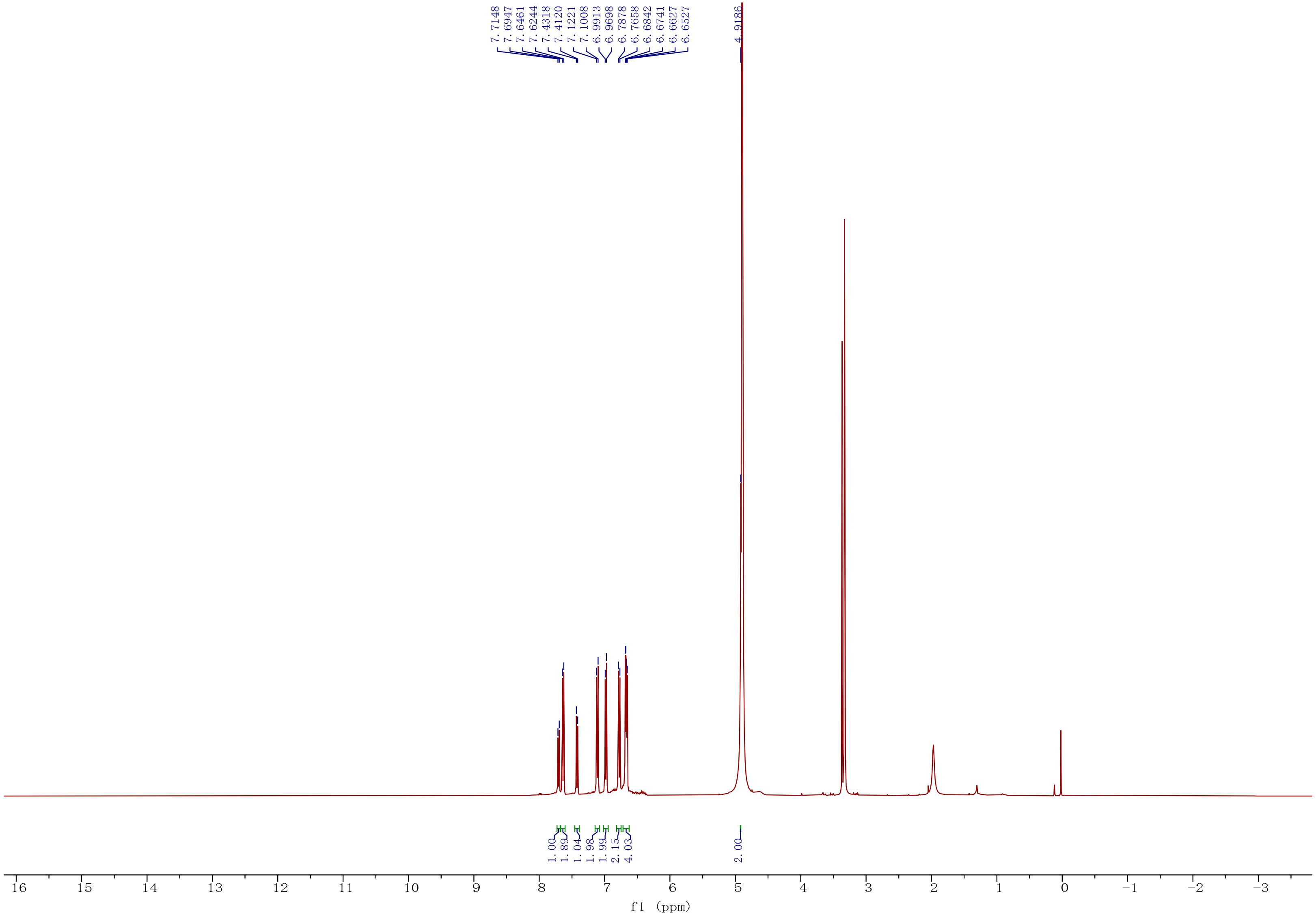

Figure S22: ^{1}H NMR spectrum of **5** in MeOH-d_4 (400 MHz).

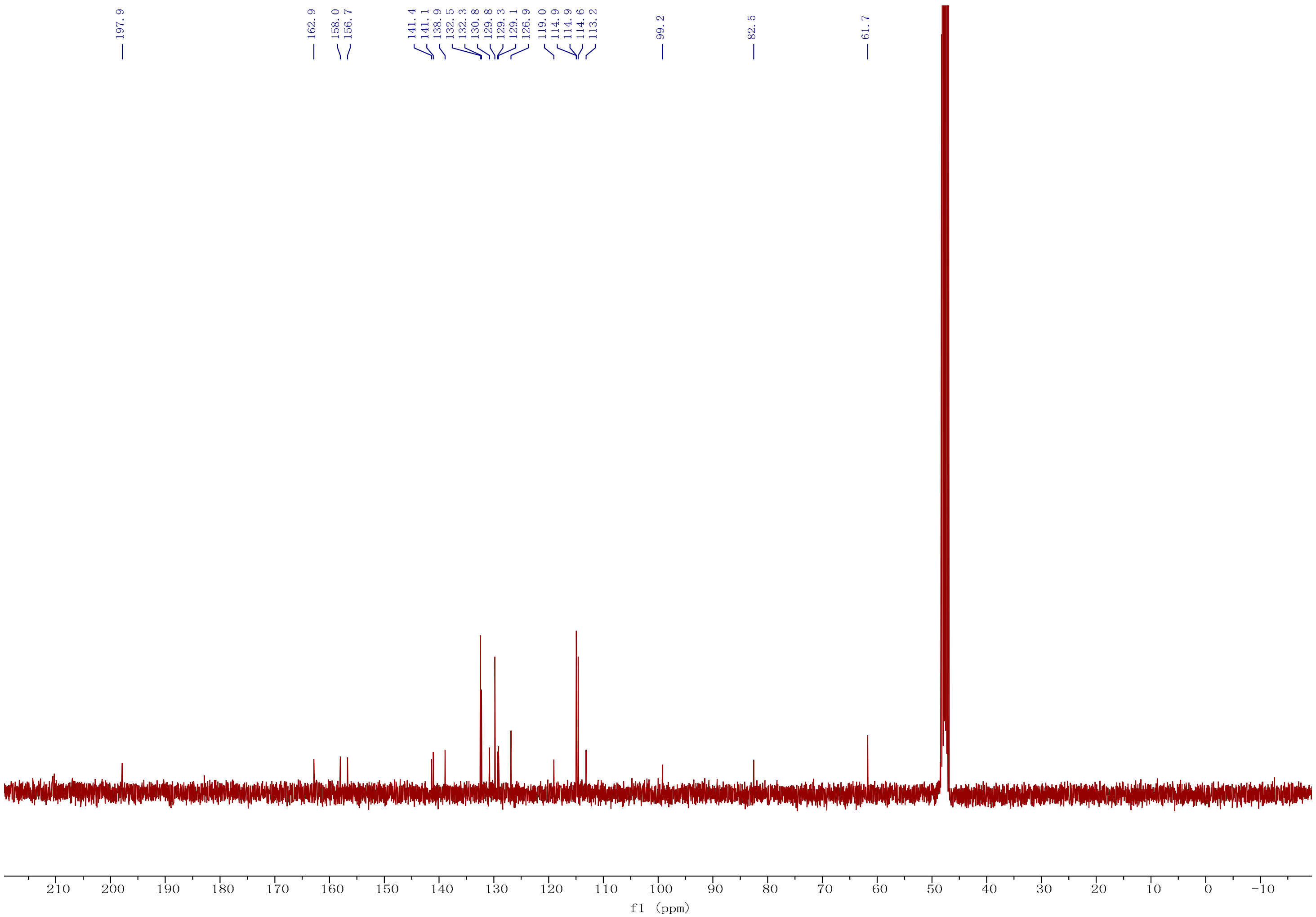

Figure S23: ^{13}C NMR spectrum of **5** in MeOH-d_4 (100 MHz).

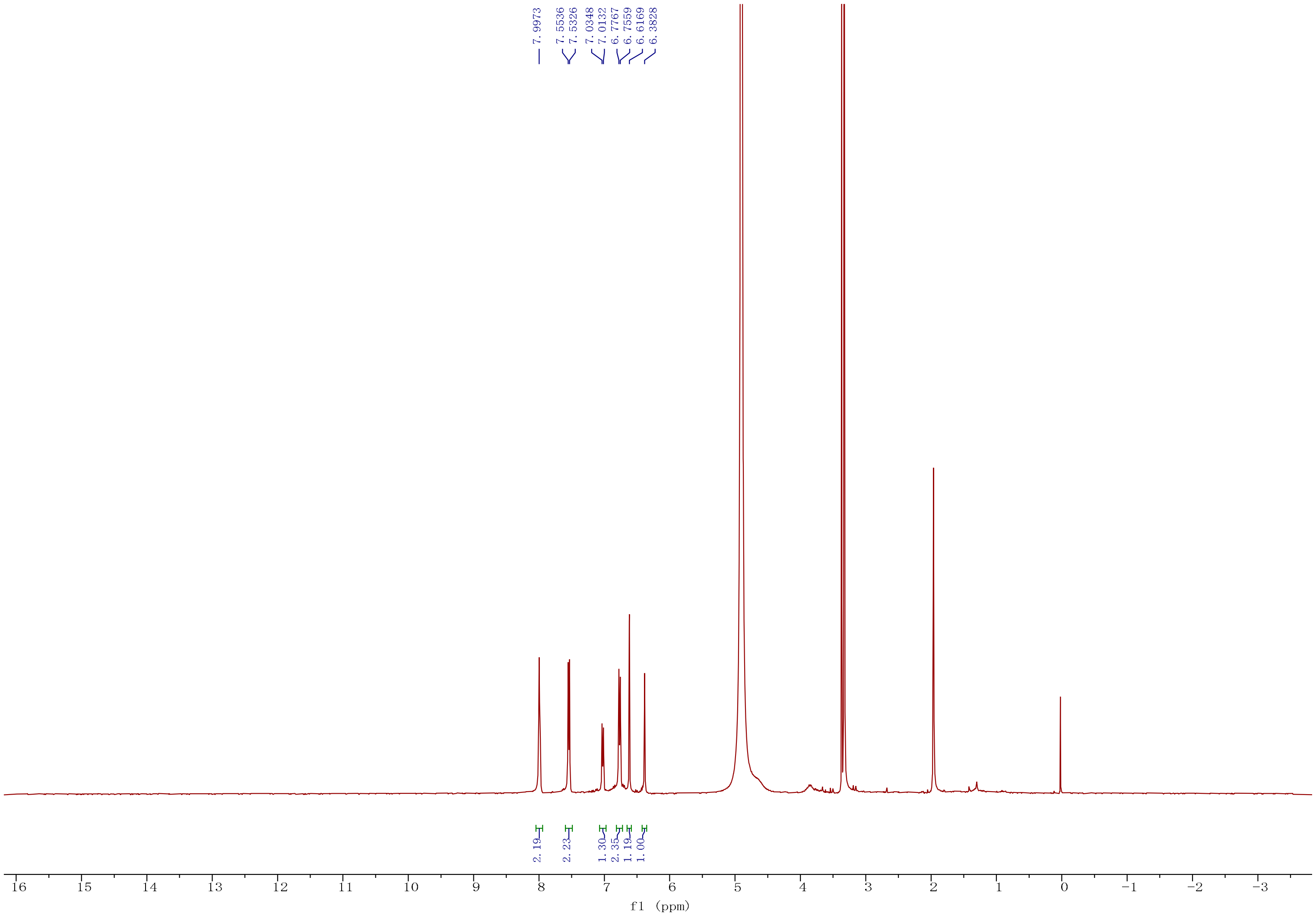

Figure S24: ^{1}H NMR spectrum of **6** in MeOH-d_4 (400 MHz).

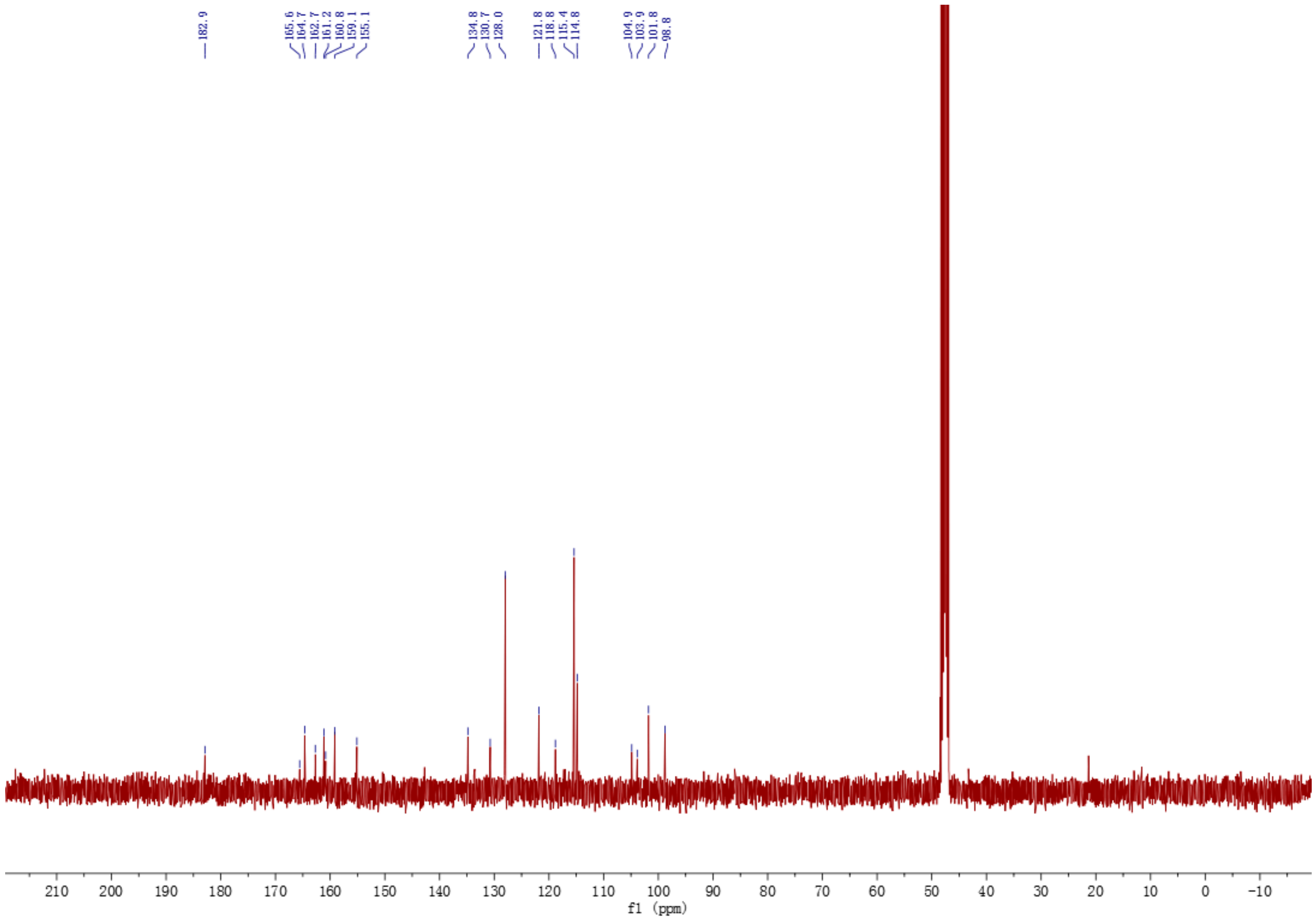

Figure S25: ^{1}H NMR spectrum of **6** in MeOH-*d*4 (400 MHz).
